# Checklist of *Epicauta* Dejean from America (Meloidae, Meloinae, Epicautini)

**DOI:** 10.3897/zookeys.807.23375

**Published:** 2018-12-17

**Authors:** María Paula ampos-Soldini, Melania Edith Safenraiter, Leandro Sebastián Wagner, Estrella Natalín ernández, Christian Javier Sequin

**Affiliations:** 1 Laboratorio de Entomología CICyTTP-CONICET/FCYT-UADER, Diamante, Entre Ríos, Argentina Laboratorio de Entomología Diamante Argentina

**Keywords:** blister beetles, Epicautini, new records, review

## Abstract

This paper presents a comprehensive list of American *Epicauta*. Two hundred and sixty-four named species were recorded in this checklist based primarily on literature and museum records. Seventy-two species were included in the subgenus Macrobasis and 192 species into the nominotypic subgenus. Nomenclatural modifications are provided for *E.langei* Borchmann and *E.nigripes* Borchmann, both junior synonyms of *E.pilme* (Molina); *E.albicincta* Borchmann, junior synonym of *E.suturalis* (Haag-Rutenberg); *E.lugubris* Denier, junior synonym of *E.tristis* (Mäklin); and *E.wagneri* Pict, junior synonym of *E.vicina* (Haag-Rutenberg). Three hundred and ninety-two new records for South America are provided.

## Introduction

Reviews of American *Epicauta* Dejean (1834) were compiled by several authors in different contributions (Werner 1945, 1954, 1955, 1957, 1958, 1973; Kaszab 1953, 1969; Pinto 1975, 1980, 1984, 1999; Adams and Selander 1979; Selander 1982; Selander and Mathieu 1969; Ballmer 1980). The most recent reviews of North and Central America were compiled by Pinto (1999) and García-París et al. (2007) and they listed a total of 173 and 124 respectively. There are no contributions for South America that include *Epicauta* in a catalogue. The reviews are gathered mostly from works dating to the last century, such as Berg 1881, 1883; Burmeister 1881; Horn 1873; Brèthes 1910, 1915; Borchmann 1917, 1930, 1937, 1940; Denier 1933a, b, 1934, 1935a, b, 1940; Pic 1928; Blackwelder 1945; Werner 1945, 1954, 1955; Martínez 1951, 1952, 1954, 1955, 1958, 1959, 1992; MacSwain 1956; Adams and Selander 1979; [Bibr B54][Bibr B55], 1982, 1984, 1991; Selander and Mathieu 1969; Agafitei and Selander 1980; Kaszab 1959, 1960; 1963a, b; and Pinto 1975, 1980, 1984, 1991. In fact, the South American species are listed in the old catalogue of Blackwelder (1945) which includes others North and Central American *Epicauta* species. The need to verify and update the checklist of Americas *Epicauta* became evident after the authors presented numerous new records for South America and saw the need to include all species of *Epicauta* in a single and updated catalogue.

## Materials and methods

The species of *Epicauta* were arranged in the order of the reviews of Pinto (1991) and the distribution data were compiled analysing all the available publications and museum data from the following institutions: Museo de La Plata, La Plata, Argentina (**MLPA**) (see Campos-Soldini et al. 2009), Instituto y Fundación Miguel Lillo, Tucumán, Argentina (**IMLA**), Museo de Ciencias Naturales “Florentino Ameghino” (**MCNFA**), and Museo Argentino de Ciencias Naturales ‘Bernardino Rivadavia’, Buenos Aires, Argentina (**MACN**), and the private collection of Barriga-Tuñón.

For seasonal distribution and information on larval stages see Selander and Weddle (1969), Pinto (1980, 1991), Bologna (1991), and Pinto and Bologna (1999). For identification keys to *Macrobasis* and the nominotypical subgenus from North and Central America see Pinto (1991). For host plants and identification keys to *E.bella* group, *E.vittata* group, and *E.maculata* group from South America see Campos-Soldini (2011), Campos-Soldini and Roig-Juñent (2011), and Campos-Solidni and Roig-Juñent (2015), respectively. Junior synonyms are provided for each species when applicable.

## Results

**Figure 1.**
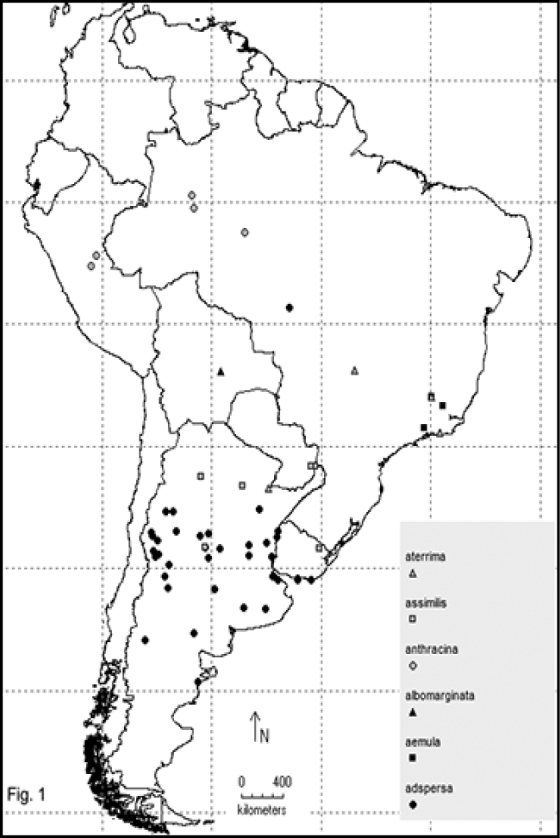
New distribution records for: *E.adspersa*; *E.aemula*; *E.albomarginata*; *E.anthracina*; *E.assimilis*; and *E.aterrima*.

**Figure 2.**
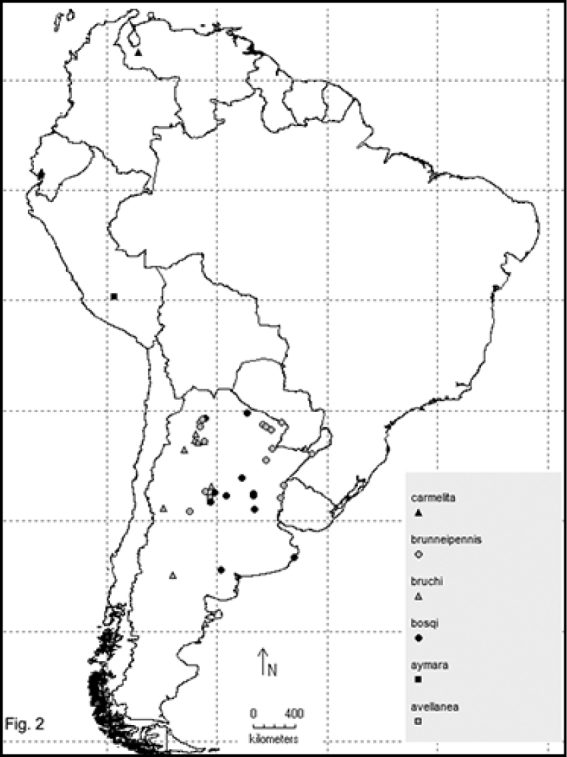
New distribution records for: *E.avellanea*; *E.aymara*; *E.bosqi*; *E.bruchi*; *E.brunneipennis*; and *E.carmelita*.

**Figure 3.**
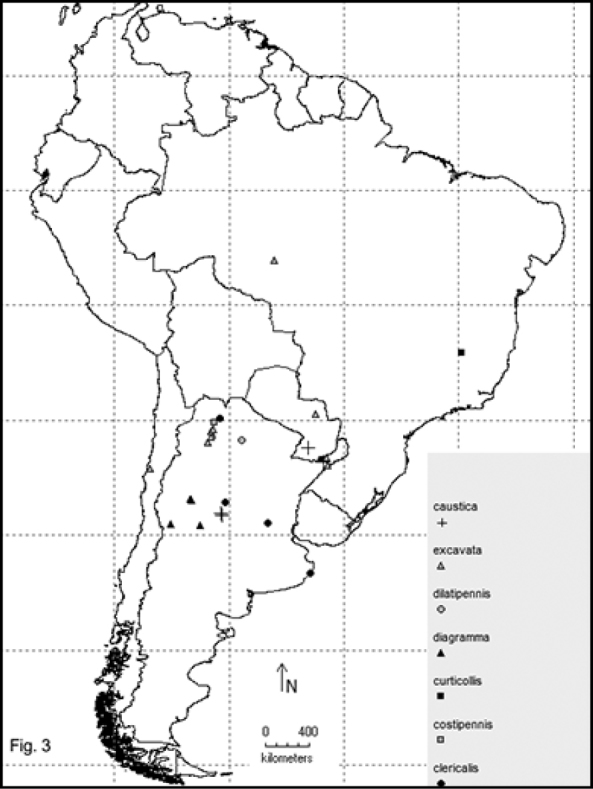
New distribution records for: *E.clericalis*; *E.costipennis*; *E.curticollis*; *E.diagramma*; *E.dilatipennis*; and *E.excavata*.

**Figure 4.**
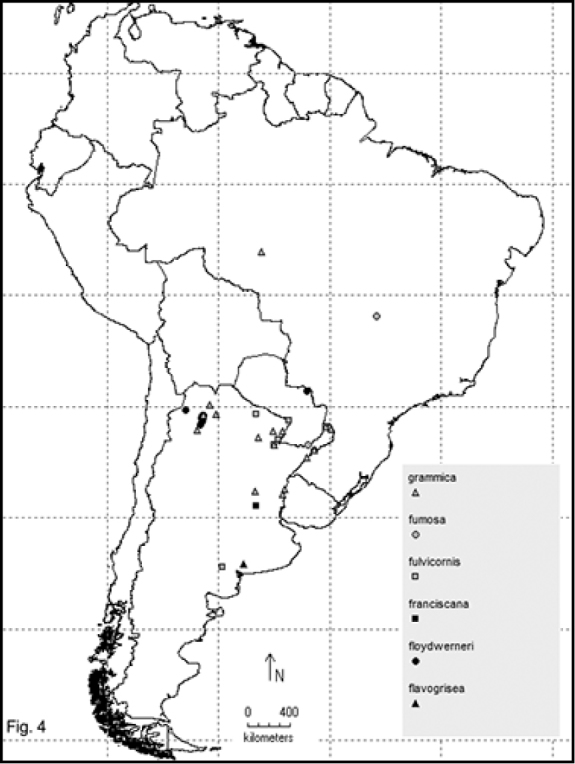
New distribution records for: *E.flavogrisea*; *E.floydwerneri*; *E.franciscana*; *E.fulvicornis*; *E.fumosa*; and *E.grammica*.

**Figure 5.**
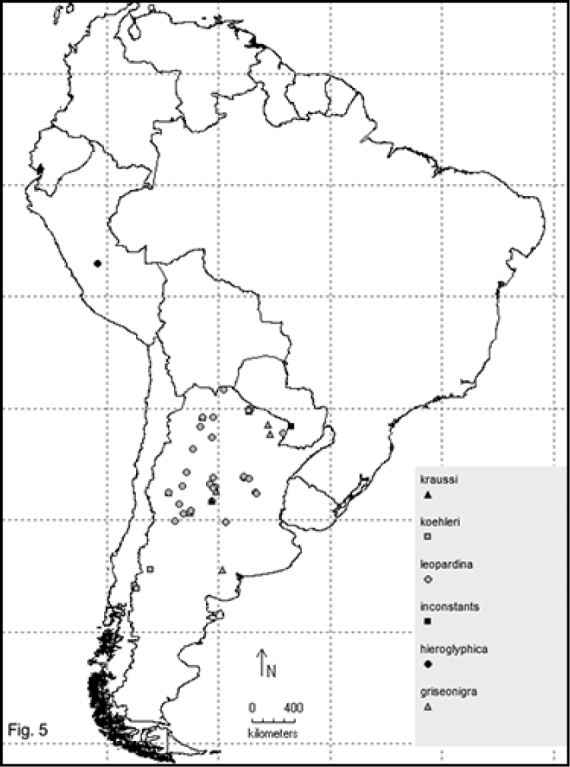
New distribution records for: *E.griseonigra*; *E.hieroglyphica*; *E.inconstants*; *E.leopardina*; *E.koehleri*; and *E.kraussi*.

**Figure 6.**
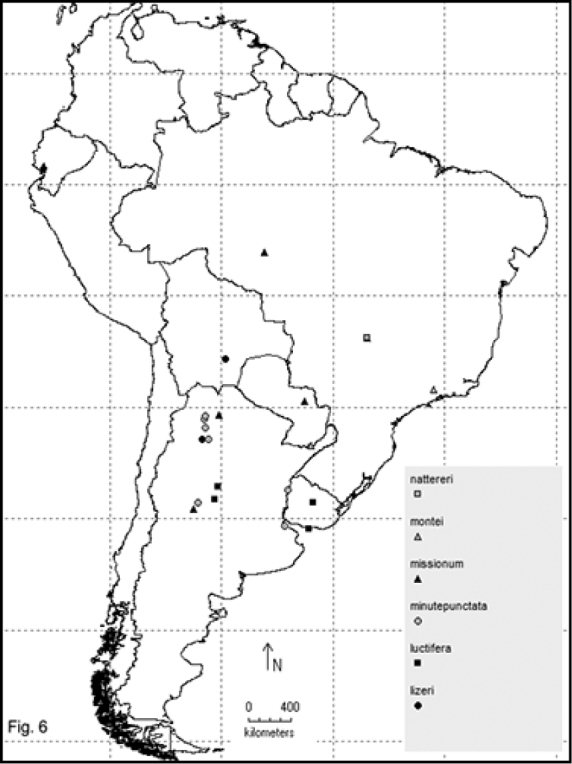
New distribution records for: *E.lizeri*; *E.luctifera*; *E.minutepunctata*; *E.missionum*; *E.montei*; and *E.nattereri*.

**Figure 7.**
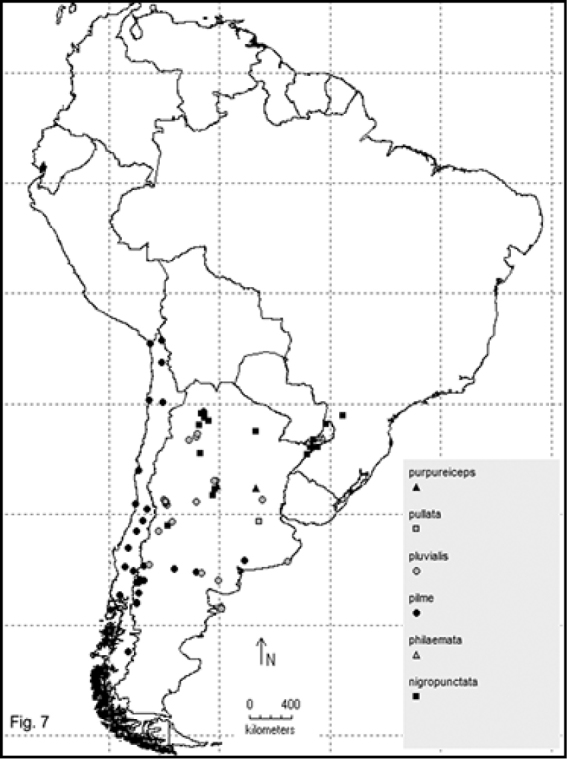
New distribution records for: *E.nigropunctata*; *E.philaemata*; *E.pilme*; *E.pluvialis*; *E.pullata*; and *E.purpureiceps*.

**Figure 8.**
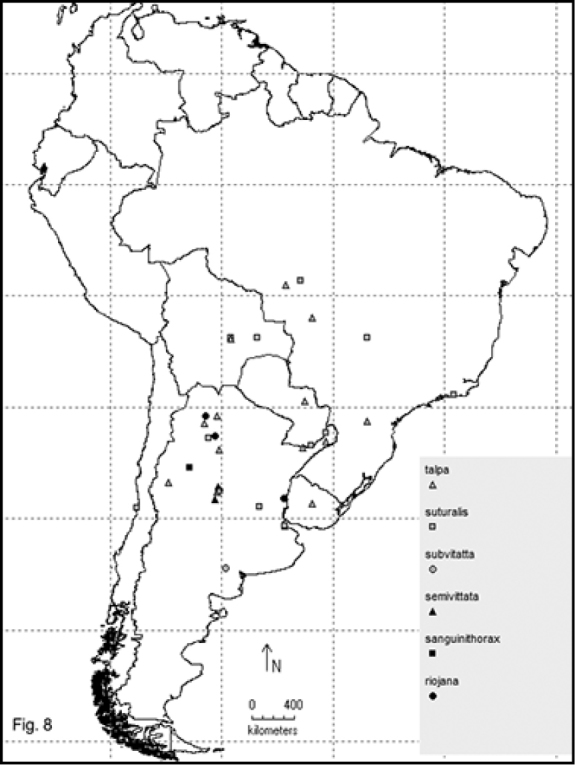
New distribution records for: *E.riojana*; *E.sanguinithorax*; *E.semivittata*; *E.subvittata*; *E.suturalis*; and *E.talpa*.

**Figure 9.**
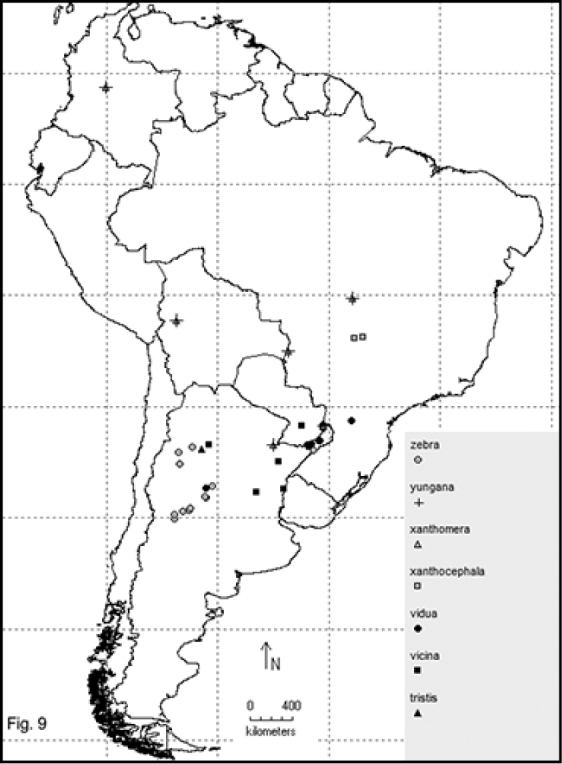
New distribution records for: *E.tristis*; *E.vicina*; *E.vidua*; *E.xanthocephala*; *E.xanthopterus*; *E.yungana*; and *E.zebra*.

Two hundred and sixty- four named species are recorded in this checklist based primarily on the literature and museum records. Seventy- two species are included in the subgenus Macrobasis and 192 species into the nominotypic subgenus. Nomenclatural modifications are provided for *E.langei* Borchmann and *E.nigripes* Borchmann junior synonym of *E.pilme* (Molina), *E.albicincta* Borchmann junior synonym of *E.suturalis* (Haag-Rutenberg), *E.lugubris* Denier junior synonym of *E.tristis* (Mäklin) and *E.wagneri* Pict junior synonym of *E.vicina* (Haag-Rutenberg). Three hundred and ninety-two new records for South America are provided for the following species: *E.adspersa* (Klug); *E.aemula* (Fischer); *E.albomarginata* (Mäklin); *E.anthracina* (Erichson); *E.assimilis* (Haag-Rutembreg); *E.aterrima* (Klug); *E.avellanea* Denier; *E.aymara* Denier; *E.bosqi* Denier; *E.bruchi* Borchmann; *E.brunneipennis* (Haag-Rutembreg); *E.carmelita* (Haag-Rutenberg); *E.caustica* Rojas; *E.clericalis* (Berg); *E.costipennis* Borchmann; *E.curticollis* Borchmann; *E.diagramma* (Burmeister); *E.excavata* (Klug); *E.flavogrisea* (Burmeisteir); *E.floydwerneri* Martínez; *E.franciscana* Denier; *E.fulvicornis* (Burmeister); *E.fumosa* (Germar); *E.grammica* (Fischer); *E.griseonigra* (Fairmaire); *E.hieroglyphica* (Haag-Rutenberg); *E.inconstants* (Fischer); *E.koehleri* Denier; *E.kraussi* (Haag-Rutenberg); *E.leopardina* (Hagg-Rutenberg); *E.lizeri* Denier; *E.luctifera* (Fairmaire); *E.minutepunctata* Borchmann; *E.missionum* (Berg); *E.montei* Denier; *E.nattereri* Haag-Rutenberg; *E.nigropunctata* Blanchard); *E.philaemata* (Klug); *E.pilme* Molina; *E.pluvialis* Borchmann; *E.pullata* (Berg); *E.purpureiceps* (Berg); *E.riojana* (Fairmaire); *E.rutilifrons* Borchmann; *E.sanguinithorax* (Haag-Rutenberg); *E.semivittata* (Fairmaire); *E.subvittata* (Erichson); *E.suturalis* (Haag-Rutenberg); *E.talpa* (Haag-Rutenberg); *E.tristis* (Mäklin); *E.vicina* (Haag-Rutenberg); *E.vidua* (Klug); *E.xanthomera* (Fischer); *E.xanthocephala* (Klug); *E.yungana* Denier; and *E.zebra* (Dohrn) (Figs 1–9). *Epicautarutilifrons* is the only species with its new records not mapped because it did not have reference points.

## Checklist

### Epicautini Denier, 1935

#### *Epicauta* Dejean, 1834

**^†^***Epicautasanctoruensis* Zaragoza-Caballero & Velasco-de León, 2003

##### Species of Epicauta (Maculata)

**Epicauta (Macrobasis) alastor Skinner, 1904**

*Epicautaalastor* Skinner, 1904: 217; Werner 1945: 436; 1954: 27; Werner et al. 1966: 46; Selander and Mathieu 1969: 15; Pinto 1991: 69.

**Distribution.** Mexico, United States.

**Location.** Mexico: Sinaloa, Sonora. United States: Arizona, California, Texas.

**Epicauta (Macrobasis) albida (Say, 1824)**

*Lyttaalbida* Say, 1824: 305.

*Lyttaluteicornis* LeConte, 1854: 84.

*Cantharisalbida*: Gemminger and Harold 1870: 2147.

*Macrobasisalbida*: LeConte, 1863–66: 68; Horn 1873: 89; Snow 1879: 69; Dugès 1889: 57; Champion 1891–93: 397; Chittenden 1903: 26; Fall and Cockerell 1907: 209.

*Epicautaalbida*: Denier 1935: 152; Werner 1945: 511; Blackwelder 1945: 482; Dillon 1952: 403; MacSwain 1956: 55; Parker and Wakeland 1957: 26; Selander and Mathieu 1969: 110; Pinto 1991: 69.

**Distribution.** Mexico, United States.

**Location.** Mexico: Coahuila, Nuevo León, Tamaulipas. United States: Arizona, Colorado, Kansas, Nebraska, New Mexico, Oklahoma, Texas, Wyoming.

**Epicauta (Macrobasis) alpina Werner, 1944**

*Epicautaalpina* Werner, 1944: 67; Werner et al. 1966: 49; Pinto 1991: 80.

**Distribution.** United States.

**Location.** United States: Arizona, New Mexico, Texas.

**Epicauta (Macrobasis) apicalis Dugès, 1889**

*Epicautaapicalis* Dugès, 1889: 410; Denier 1935: 152; Blackwelder 1945: 482; Pinto 1991: 75.

**Distribution.** Mexico.

**Location.** Mexico: Nayarit.

**Epicauta (Macrobasis) arizonica Werner, 1944**

*Epicautaarizonica* Werner, 1944: 72; 1966: 48; Selander 1982: 797; Pinto 1991: 71.

**Distribution.** Mexico, United States.

**Location.** Mexico: Baja California Sur, Chihuahua, Nayarit, Sonora. United States: Arizona.

**Epicauta (Macrobasis) atricolor Champion, 1892**

*Epicautaatricolor* Champion, 1892: 419; Denier 1935: 152; Blackwelder 1945: 482; Werner 1954: 27; 1958: 1; Pinto 1982: 405; 1991: 80; 1991: 80.

**Distribution.** Mexico.

**Location.** Mexico: Morelos, Oaxaca, Veracruz.

**Epicauta (Macrobasis) atripilis Champion, 1892**

*Epicautaatripilis* Champion, 1892: 410; Denier 1935: 152; Blackwelder 1945: 482; Pinto 1991: 75.

**Distribution.** Mexico.

**Location.** Mexico: Oaxaca, Veracruz.

**Epicauta (Macrobasis) atrivittata (LeConte, 1854)**

*Lyttaatrivittata* LeConte, 1854: 224.

*Macrobasisatrivittis* [sic]: LeConte 1863–66: 68 (lapsus calami).

*Cantharisatrivittata*: Gemminger and Harlod 1870: 2148.

*Macrobasisatrivittata*: Horn 1873: 90; Snow 1907: 186; Fall and Cockerell 1907: 209.

*Epicautaatrivittata*: Denier 1935: 152; Selander and Mathieu 1969: 88; Pinto 1991: 70.

**Distribution.** Mexico, United States.

**Location.** Mexico: Chihuahua, Coahuila, Durango. United States: Arizona, New Mexico, Texas.

**Epicauta (Macrobasis) balli Werner, 1945**

*Epicautaballi* Werner, 1945: 460; Werner et al. 1966: 34; Pinto 1991: 81.

**Distribution.** United States.

**Location.** United States: Arizona.

**Epicauta (Macrobasis) bekeri (Dugès, 1889)**

*Epicautabekeri* Dugès, 1889: 113.

*Macrobasisbekeri*: Champion 1892: 400.

*Epicautabekeri*: Denier 1935: 153; Blackwelder 1945: 482; Werner 1949: 76; Pinto 1991: 72.

**Distribution.** Mexico.

**Location.** Mexico: Colima, Durango, Morelos, Nayarit.

**Epicauta (Macrobasis) bipunctata Werner, 1958**

*Epicautabipunctata* Werner, 1958: 7; Selander and Ahafitei 1982: 200; Pinto 1991: 81.

**Distribution.** Mexico.

**Location.** Mexico: Guerrero, Jalisco, Morelos.

**Epicauta (Macrobasis) borrei Werner, 1958**

*Lyttafumosa* Haag-Rutenberg, 1880: 40, nec Germar, 1824.

*Cantharisborrei* Dugès, 1881: 145.

*Macrobasisborrei* Horn, 1885: 107.

*Macrobasisfumosa*: Champion 1899: 178.

*Epicautaborrei*: Denier 1935: 153; Blackwelder 1945: 482; Pinto 1991: 81.

**Distribution.** Mexico.

**Location.** Mexico: Aguascalientes, Durango, Guanajuato, Jalisco, Mexico, Michoacán, Zacatecas.

**Epicauta (Macrobasis) candezi (Haag-Rutenberg, 1880)**

*Lyttacandezi* Haag-Rutenberg, 1880: 43.

*Epicautadiversicornis*: Champion 1892: 399 (in part).

*Epicautacandezi*: Werner 1949: 76; Pinto 1991: 72.

**Distribution.** El Salvador, Guatemala, Mexico.

**Location.** El Salvador and Guatemala: country labeled only. Mexico: Chiapas, Yucatán.

**Epicauta (Macrobasis) cinereiventris Champion, 1892**

*Epicautacinireiventris* Champion, 1892: 411; Denier 1935: 154; Blackwelder 1945: 483; Pinto 1991: 75.

**Distribution.** Mexico.

**Location.** Mexico: Chiapas, Guerrero.

**Epicauta (Macrobasis) croceicincta Dugès, 1881**

*Canthariscroceicincta* Dugès, 1881: 143; Champion 1892: 42.

*Epicautacroceicincta*: Denier 1935: 154; Blackwelder 1945: 483; Werner 1954: 27; Pinto 1991: 75.

**Distribution.** Mexico.

**Location.** Mexico: Guanajuato, Jalisco.

**Epicauta (Macrobasis) disparilis (Champion, 1892)**

*Macrobasisdisparilis* Champion, 1892: 398.

*Epicautadisparilis*: Denier 1935: 154; Blackwelder 1945: 483; Werner 1973: 463; Pinto 1991: 78.

**Distribution.** Mexico.

**Location.** Mexico: Guerrero, Puebla.

**Epicauta (Macrobasis) distorta (Champion, 1892)**

*Macrobasisdistorta* Champion, 1892: 396.

*Epicautadistorta*: Denier 1935: 154; Blackwelder 1945: 483; Werner 1973: 463; Pinto 1991: 79.

**Distribution.** Costa Rica, Honduras, Mexico, Nicaragua.

**Location.** Mexico: Guerrero.

**Epicauta (Macrobasis) diversicornis (Haag-Rutenberg, 1880)**

*Lyttadiversicornis* Haag-Rutenberg, 1880: 42.

*Lyttapallida* Haag-Rutenberg, 1880: 42.

*Macrobasisflavens* Dugès, 1889: 58.

*Epicautadiversicornis*: Denier 1935: 154; Blackwelder 1945: 483; Werner 1949: 76; Pinto 1991: 72.

**Distribution.** Mexico.

**Location.** Mexico: Guerrero, Hidalgo, Jalisco, Mexico, Michoacán, Morelos, Sinaloa, Sonora.

**Epicauta (Macrobasis) dohrni (Haag-Rutenberg, 1880)**

*Lyttadohrni* Haag-Rutenberg, 1880: 45.

*Lyttabimaculosa* Kirsch, 1886: 336.

*Lyttabogotensis* Pic, 1916: 9.

*Epicautadohrni*: Champion 1892: 409; Denier 1935: 22; Blackwelder 1945: 483; Pinto 1991: 73.

**Distribution.** Colombia, Panama: country labeled only.

**Epicauta (Macrobasis) evanescens Champion, 1892**

*Epicautaevanescens* Champion, 1892: 412; Denier 1935: 155; Blackwelder 1945: 483; Pinto 1991: 76.

**Distribution.** Guatemala, Mexico.

**Location.** Guatemala: country labeled only. Mexico: Chiapas.

**Epicauta (Macrobasis) excors (Fall, 1909)**

*Macrobasisexcors* Fall, 1909: 166.

*Epicautaexcors*: Denier 1935: 155; Werner 1945: 503; Pinto 1991: 79.

**Distribution.** Mexico.

**Location.** Mexico: Baja California Sur.

**Epicauta (Macrobasis) fabricii (LeConte, 1853)**

*Lyttacinerea* Fabricius, 1798: 119, nec *Meloecinereous* Forster, 1771.

*Lyttafabricii* LeConte, 1853: 343.

*Lyttadebilis* LeConte, 1853: 344.

*Cantharisfabricii*: Gemminger and Harold 1980: 2150.

*Epicautamurinoides* Dillon, 1952: 409.

*Epicautafabricii*: Werner et al. 1966: 46; Selander 1982: 820; Pinto 1991: 74.

**Distribution.** Canada, United States.

**Location.** Canada: Manitoba. United States: Arizona, Colorado, Kansas, Montana, Nebraska, North Dakota, Oklahoma, South Dakota, Texas, Utah.

**Epicauta (Macrobasis) flagellaria (Erichson, 1848)**

*Lyttaflagellaria* Erichson, 1848: 566.

*Cantharisflagellaria*: Gemminger and Harold 1870: 2150.

*Lyttaintermedia* Haag-Rutenberg, 1880: 56.

*Epicautaintermedia*: Blackwelder 1945: 483.

*Epicautaflagellaria*: Blackwelder 1945: 483; Werner 1949: 76; Pinto 1991: 72.

**Distribution.** Colombia: Barranquilla, Tolima, Panama, Trinidad, Venezuela.

**Epicauta (Macrobasis) flavocinerea (Blatchely, 1910)**

*Macrobasisflavocinereus* Blatchely, 1910: 1359.

*Epicautaflavocinerea*: Denier 1935: 155; Werner 1945: 499; Pinto 1991: 74.

**Distribution.** Canada, United States.

**Location.** Canada: New Brunswick. United States: Colorado, Iowa, Maine, Massachusetts, Michigan, Minnesota, Montana, Nebraska, New Hampshire, North Dakota, South Dakota, Wisconsin, Wyoming.

**Epicauta (Macrobasis) forticornis (Haag-Rutenberg, 1880)**

*Lyttaforticornis* Haag-Rutembeerg, 1880: 41.

*Epicautaforticornis*: Denier 1935: 155; Blackwelder 1945: 483; Werner 1949: 75; Pinto 1991: 72.

**Distribution.** Mexico.

**Location.** Mexico: Guerrero, Morelos, Nayarit, Yucatán.

**Epicauta (Macrobasis) funesta (Chevrolat, 1834)**

*Lyttafunesta* Chevrolat, 1834: 3.

*Cantharisfunesta*: Gemminger and Harold 1870: 2150.

*Epicautafunesta*: Champion 1892: 410; Denier 1935: 155; Blackwelder 1945: 483; Selander and Agafitei 1982: 200; Pinto 1991: 76.

**Distribution.** Mexico.

**Location.** Mexico: Puebla, Veracruz.

**Epicauta (Macrobasis) gissleri (Horn, 1878)**

*Macrobasisgissleri* Horn, 1878: 58.

*Epicautagissleri*: Denier 1935: 155; Werner 1945: 496; Werner et al. 1966: 52; Pinto 1991: 78

**Distribution.** United States.

**Location.** United States: Arizona, New Mexico, Texas.

**Epicauta (Macrobasis) haroldi (Haag-Rutenberg, 1880)**

*Lyttaharoldi* Haag-Rutenberg, 1880: 44.

*Epicautaharoldi*: Champion 1892: 410; Denier 1935: 156; Blackwelder 1945: 483; Werner 1958: 1; Pinto 1991: 76.

**Distribution.** Costa Rica, Guatemala.

**Epicauta (Macrobasis) hirsutipubescens (Maydell, 1934)**

*Macrobasishirustipubescens* Maydell, 1934: 334.

*Epicautavirgulata*: Werner 1945: 512 (in part).

*Epicautahirustipubescens*: Denier 1940: 420; Werner 1949: 110; Werner et al. 1966: 51; Pinto 1991: 83.

**Distribution.** Mexico, United States.

**Location.** Mexico: Chihuahua, Coahulia, Durango, Sonora. United States: Arizona, New Mexico, Texas.

**Epicauta (Macrobasis) humeralis (Dugès, 1889)**

*Macrobasishumeralis* Dugès, 1889: 58; Champion, 1892: 400.

*Epicautahumeralis*: Denier 1935: 156; Werner 1949: 76; Pinto 1991: 72.

**Distribution.** Mexico.

**Location.** Mexico: Morelos, Jalisco, Nayarit.

**Epicauta (Macrobasis) immaculata (Say, 1824)**

*Lyttaimmaculate* Say, 1824: 304.

*Lyttaarticularis* Say, 1824: 304.

*Lyttafulvescens* LeConte, 1854: 447.

*Cantharisfulvescens*: Gemminger and Harold 1870: 2150.

*Cantharisimmaculata*: Gemminger and Harold 1870: 2151.

*Macrobasisfulvescens*: LeConte 1863-66: 68; Snow 1879: 69.

*Macrobasisimmaculate*: LeConte 1863-66: 68; Horn 1873: 93; Ulke 1875: 862; Chittenden 1903: 26; Blatchley 1910: 1359; Milliken 1921: 7; Hatch and Ortenburger 1930: 13; Böving and Craighead 1931: 96; Carruth 1931: 53; Gilberston and Horsfall 1940: 5; Montgomery and Amos 1941: 254; Schwitzgebel and Wilbur 1942: 42.

*Cantharisbasalis* Dugès, 1881: 144; 1889: 71.

*Macrobasisbasalis*: Champion 1892: 402.

*Epicautaimmaculata*: Denier 1935: 156; Werner 1945: 489; Dillon 1952: 407; MacSwain 1956: 52; Selander and Mathieu 1969: 113; Pinto 1991: 70.

**Distribution.** Mexico, United States.

**Location.** Mexico: Coahuila, Tamaulipas, Veracruz. United States: Alabama, Arkansas, Colorado, Georgia, Illinois, Indiana, Iowa, Kansas, Kentucky, Louisiana, Mississippi, Missouri, Nebraska, New Mexico, Ohio, Oklahoma, South Dakota, Tennessee, Texas, West Virginia.

**Epicauta (Macrobasis) ingrata Fall, 1907**

*Epicautaingrata* Fall, 1907: 258; Denier 1935: 156; Werner et al. 1966: 45; Pinto 1991: 77

**Distribution.** United States.

**Location.** United States: Arizona, Colorado, New Mexico.

**Epicauta (Macrobasis) isthmica Werner, 1949**

*Epicautaisthmica* Werner, 1949: 72; Pinto 1991: 73.

**Distribution.** Belize, Costa Rica, Honduras, Mexico, Nicaragua, Panama.

**Location.** Belize, Costa Rica, Hondura, Nicaragua, and Panama. Mexico: Querétaro, Veracruz.

**Epicauta (Macrobasis) labialis (Dugès, 1881)**

*Cantharislabialis* Dugès, 1881: 145.

*Macrobasislabialis*: Dugès 1889: 86.

*Ganospastalabialis*: Champion 1892: 403.

*Epicautalabialis*: Selander 1954: 86; Werner 1958: 19; Pinto 1991: 81.

**Distribution.** Mexico.

**Location.** Mexico: Guanajuato, Jalisco.

**Epicauta (Macrobasis) languida (Horn, 1895)**

*Macrobasislanguida* Horn, 1895: 252.

*Epicautalanguida*: Denier 1935: 156; Blackwelder 1945: 483; Werner 1945: 501; 1954: 113; Pinto 1991: 90.

**Distribution.** Mexico.

**Location.** Mexico: Baja California Sur.

**Epicauta (Macrobasis) lauta (Horn, 1885)**

*Macrobasislauta* Horn, 1885: 108.

*Epicautacompressicollis* Champion, 1892: 427.

*Epicautamacroflexi* Dillon, 1952: 414.

*Epicautalauta*: Denier 1935: 156; Vaurie 1950: 86; Werner 1954: 27; Werner et al. 1966: 47; Selander 1982: 797; Pinto 1991: 78.

*Epicautalautarossi* Werner, 1949: 108.

*Epicautalautalauta*: Selander 1954: 86.

**Distribution.** Mexico, United States.

**Location.** Mexico: Baja California Sur, Chihuahua, Coahuila, Durango. United States: Arizona, California, New Mexico, Texas.

**Epicauta (Macrobasis) leoni Dugès, 1889**

*Epicautaleoni* Dugès, 1889: 15; Denier 1935: 156; Blackwelder 1945: 483; Werner 1958: 15; Selander and Agafitei 1982: 200; Pinto 1991: 82.

**Distribution.** Mexico.

**Location.** Mexico: Michoacán, Nuevo León, San Luis Potosí, Tamaulipas.

**Epicauta (Macrobasis) liebecki Werner, 1944**

*Epicautaliebecki* Werner, 1944: 72; Werner et al. 1966: 49; Pinto 1991: 73.

**Distribution.** United States.

**Location.** United States: Arizona.

**Epicauta (Macrobasis) linearis (LeConte, 1858)**

*Lyttalinearis* LeConte, 1858: 23.

*Epicautalinearis*: Denier 1935: 156; Werner 1945: 513; Selander 1954: 86; Werner et al. 1966: 56; Pinto 1991: 83.

**Distribution.** Mexico, United States.

**Location.** Mexico: Chihuahua, Durango. United States: Arizona, New Mexico, Texas.

**Epicauta (Macrobasis) longicollis (LeConte, 1853)**

*Lyttalongicollis* LeConte, 1853: 343.

*Macrobasislongicollis*: LeConte 1863-66: 68; Horn 1873: 90; Champion 1891-93: 397; Snow 1906: 174; Fall and Cockerell 1907: 209; Cockerell and Harris 1925: 31.

*Cantharislongicollis*: Gemminger and Harold 1870: 2151.

*Epicautalongicollis*: Denier 1935: 156; 1940: 421; Blackwelder 1945: 483; Werner 1945: 508; Vaurie 1950: 35; Dillon 1952: 406; MacSwain 1956: 56; Selander and Mathieu 1969: 109; Pinto 1991: 71.

**Distribution.** Mexico. United States.

**Location.** Mexico: Chihuahua, Coahuila. United States: Arizona, Colorado, Missouri, New Mexico, Texas.

**Epicauta (Macrobasis) maculifera (Maydell, 1934)**

*Macrobasismaculifera* Maydell, 1934: 335.

*Epicautamaculifera*: Denier 1940: 421; Werner 1945: 514; 1973: 463; Werner et al. 1966: 50; Pinto 1991: 79.

**Distribution.** United States.

**Location.** United States: Arizona.

**Epicauta (Macrobasis) melanochroa Wellman, 1910**

*Cantharisnigra* Dugès, 1870: 161.

*Epicautanigra*: Dugès 1889: 76, nec Woodhouse 1800.

*Epicautamelanochroa* Wellman, 1910: 24 (n. repl. name); Werner 1958: 11; Pinto 1991: 82

**Distribution.** Mexico.

**Location.** Mexico: Guanajuato, Jalisco, Mexico, Michoacán, Morelos, Nayarit, Querétaro.

**Epicauta (Macrobasis) mimetica (Horn, 1875)**

*Cantharismimetica* Horn, 1875: 154.

*Epicautamimetica*: Werner 1945: 453; 1958: 17.

**Distribution.** United States.

**Location.** United States: Texas.

**Epicauta (Macrobasis) murina (LeConte, 1853)**

*Cantharisunicolor* Kirby, 1837: 241, nec *Cantharisunicolor* Faldermann, 1835.

*Lyttamurina* LeConte, 1853: 344.

*Cantharismurina*: Gemminger and Harold 1870: 2152.

*Epicautamurina*: Werner 1945: 499; Pinto 1991: 74.

**Distribution.** Canada, United States.

**Location.** Canada: New Brunswick. United States: Colorado, Iowa, Maine, Massachusetts, Michigan, Minnesota, Montana, Nebraska, New Hampshire, North Dakota, South Dakota, Wisconsin, Wyoming.

**Epicauta (Macrobasis) nigritibialis Werner, 1958**

*Epicautanigritibialis* Werner, 1958: 5; Pinto 1991: 82.

**Distribution.** Mexico.

**Location.** Mexico: Coahuila.

**Epicauta (Macrobasis) niveolineata (Haag-Rutenberg, 1880)**

*Lyttaniveolineata* Haag-Rutenberg, 1880: 46.

*Epicautaniveolineata*: Denier 1935: 158; Blackwelder 1945: 483; Werner 1958: 4; Selander and Agafitei 1982: 200; Pinto 1991: 82.

**Distribution.** Mexico.

**Location.** Mexico: Chiapas, Guerrero, Oaxaca, Veracruz.

**Epicauta (Macrobasis) ochrea (LeConte, 1853)**

*Lyttaochrea* LeConte, 1853: 342.

*Cantharisochrea*: Gemminger and Harold 1870: 2152.

*Cantharisprotarsalis* Dugès, 1877: 62.

*Macrobasisochrea*: Champion 1892: 401.

*Epcicautamonoliformis* Dillon, 1950: 103.

*Epicautaochrea*: Denier 1935: 158; Blackwelder 1945: 484; Werner 1945: 495; 1954: 106; Werner et al. 1966: 52; Pinto 1991: 78.

**Distribution.** Mexico, United States.

**Location.** Mexico: Guanajuato, Sonora. United States: Arizona, Oklahoma, Texas, Utah.

**Epicauta (Macrobasis) pacifica Maydell, 1934**

*Epicautapacifica* Maydell, 1934: 330; Denier 1940: 421; Blackwelder 1945: 484; Pinto 1991: 76.

**Distribution.** Mexico.

**Location.** Mexico: Jalisco.

**Epicauta (Macrobasis) parkeri Werner, 1944**

*Epicautaparkeri* Werner, 1944: 71; 1945: 497; Werner et al. 1966: 53; Pinto 1991: 78.

**Distribution.** United States.

**Location.** United States: Arizona, Colorado, New Mexico.

**Epicauta (Macrobasis) polingi Werner, 1944**

*Epicautapolingi* Werner, 1944: 71; Werner et al. 1966: 48; Selander 1982: 804; Pinto 1991: 73.

*Epicautaluteola* Dillon, 1952: 411.

**Distribution.** Mexico, United States.

**Location.** Mexico: Chihuahua, Coahuila, Durango, Nuevo León, Tamaulipas. United States: Arizona, Texas.

**Epicauta (Macrobasis) prosopidis Werner, 1973**

*Epicautaprosopidis* Werner, 1973: 461; Pinto 1991: 79.

**Distribution.** Mexico.

**Location.** Mexico: Durango.

**Epicauta (Macrobasis) punctum (Dugès, 1870)**

*Cantharispunctum* Dugès, 1870: 158.

*Epicautapunctum*: Champion 1892: 410; Blackwelder 1945: 484; Pinto 1991: 76.

**Distribution.** Mexico.

**Location.** Mexico: Morelos, Oaxaca.

**Epicauta (Macrobasis) purpurea (Horn, 1885)**

*Macrobasispurpurea* Horn, 1885: 108.

*Epicautapurpurea*: Werner 1945: 504; 1973: 463; Werner et al. 1966: 50; Pinto 1991: 79.

**Distribution.** Mexico, United States.

**Location.** Mexico: Durango, Sinaloa, Sonora. United States: Arizona.

**Epicauta (Macrobasis) segmenta (LeConte, 1853)**

*Lyttasegmenta* LeConte, 1853: 343.

*Apterospastasegmentata*: LeConte 1862: 272; 1863-66: 68.

*Macrobasissegmenta*: Horn 1873: 93; Champion 1891-93: 401; Chittenden 1903: 26; Snow 1906: 174; 1907: 150; Carruth 1931: 54; Whelan 1939: 118.

*Macrobasiscinctothorax* Dugès, 1889: 65.

*Epicautasegmenta*: Blackwelder 1945: 484; Selander and Mathieu 1969: 116; Pinto 1991: 71.

**Distribution.** Mexico, United States.

**Location.** Mexico: Chihuahua, Coahuila, Durango, Sinaloa, Sonora. United States: Arizona, California, Kansas, Nebraska, New Mexico, Oklahoma, South Dakota, Texas, Wyoming.

**Epicauta (Macrobasis) selanderorum Werner, 1958**

*Epicautaselanderorum* Werner, 1958: 6; Pinto 1991: 82.

**Distribution.** Mexico.

**Location.** Mexico: Jalisco, Michoacán, Querétaro.

**Epicauta (Macrobasis) stigmata (Dugès, 1870)**

*Cantharisstigmata* Dugès, 1870: 159.

*Lyttaneglecta* Haag-Rutenberg, 1880: 54.

*Epicautastigmata*: Denier 1935: 159; Blackwelder 1945: 484; Werner 1958: 9; Pinto 1982, Pinto 1991: 82.

**Distribution.** Mexico.

**Location.** Mexico: Guanajuato, Jalisco, Mexico, Michoacán, Morelos, Querétaros, Puebla, Tlaxcala.

**Epicauta (Macrobasis) subglabra (Fall, 1922)**

*Macrobasissubglabra* Fall, 1922: 173.

*Epicautasubglabra*: Denier 1935: 159; Werner et al. 1966: 47; Pinto 1991: 74.

**Distribution.** Canada, United States.

**Location.** Canada: Alberta, Manitoba, Saskatchewan. United States: Arizona, Idaho, Michigan, New Mexico, North Dakota, South Dakota.

**Epicauta (Macrobasis) sublineata (LeConte, 1854)**

*Lyttasublineata* LeConte, 1854: 447.

*Cantharissublineata*: Gemminger and Harold 1870: 2154.

*Macrobasissublineata*: LeConte 1863-66: 68; Horn 1873: 94.

*Macrobasismegacephala* Champion, 1892: 402.

*Epicautareinhardi* Dillon, 1952: 413.

*Epicautasublineata*: Selander and Mathieu 1969: 116; Pinto 1991: 71.

**Distribution.** Mexico, United States.

**Location.** Mexico: Coahuila, Nuevo León, Tamaulipas. United States: Texas.

**Epicauta (Macrobasis) tenella (LeConte, 1854)**

*Lyttatenella* LeConte, 1854: 23.

*Cantharistenella*: Gemminger and Harold 1870: 2154.

*Epicautamerkeliana* Horn, 1891: 43.

*Epicautatenella*: Horn 1891: 43; Denier 1935: 159; Selander 1954: 87; Werner et al. 1966: 45; Pinto 1991: 79.

**Distribution.** Mexico, United States.

**Location.** Mexico: Baja California, Chihuahua, Durango, Sonora. United States: Arizona, California, New Mexico, Nevada, Texas.

**Epicauta (Macrobasis) tenuemarginata Werner, 1958**

*Epicautatenuemarginata* Werner, 1958: 13; Pinto 1991: 83.

**Distribution.** Mexico.

**Location.** Mexico: Jalisco, Michoacán.

**Epicauta (Macrobasis) tenuicornis (Champion, 1892)**

*Macrobasistenuicornis* Champion, 1892: 400.

*Epicautatenuicornis*: Werner 1954: 27; Pinto 1991: 73.

**Distribution.** Mexico.

**Location.** Mexico: Guerrero, Michoacan, Morelos, Puebla.

**Epicauta (Macrobasis) tenuilineata (Horn, 1894)**

*Macrobasistenuilineata* Horn, 1894: 436.

*Epicautatenuilineata*: Denier 1935: 159; Blackwelder 1945: 484; Werner 1945: 502; Werner et al. 1966: 35; Pinto 1991: 80.

**Distribution.** Mexico, United States.

**Location.** Mexico: Baja California Sur. United States: Arizona, California.

**Epicauta (Macrobasis) tenuis (LeConte, 1853)**

*Lyttatenuis* LeConte, 1853: 343.

*Cantharistenuis*: Gemminger and Harold 1870: 2154.

*Epicautatenuis*: Denier 1935: 159; 1940: 422; Werner 1945; Pinto 1991: 80.

**Distribution.** United States.

**Location.** United States: Florida, Georgia, South Carolina.

**Epicauta (Macrobasis) terminata (Dugès, 1870)**

*Cantharisterminata* Dugès, 1870: 157; Gemminger and Harold 1870: 2154.

*Epicautaterminata*: Dugès 1889: 78; Champion 1892: 409; Denier 1935: 159; Blackwelder 1945: 484; Selander 1954: 88; Pinto 1991: 77.

**Distribution.** Mexico.

**Location.** Mexico: Colima, Guerrero, Jalisco, Michoacán, Oaxaca, Puebla, Veracruz.

**Epicauta (Macrobasis) texana Werner, 1944**

*Epicautatexana* Werner, 1944: 73; Selander and Mathieu 1969: 110; Pinto 1991: 71.

**Distribution.** Mexico, United States.

**Location.** Mexico: Durango. United States: Arizona, New Mexico, Texas.

**Epicauta (Macrobasis) torsa (LeConte, 1853)**

*Lyttatorsa* LeConte, 1853: 343.

*Epicautatorsa*: Denier 1935: 159; 1940: 422; Werner 1945: 508; 1973: 463; Arnold 1976: 28; Pinto 1991: 79.

**Distribution.** United States.

**Location.** United States: Alabama, Florida, Massachusetts, Mississippi, North California, Oklahoma, Texas.

**Epicauta (Macrobasis) tripartita Champion, 1892**

*Epicautatripartita* Champion, 1892: 421; Denier 1935: 159; Blackwelder 1945: 484; Vaurie 1950: 25; Werner 1958: 4; Pinto 1991: 83.

**Distribution.** Mexico.

**Location.** Mexico: Chihuahua, Nayarit, Sinaloa.

**Epicauta (Macrobasis) triquetra Werner, 1958**

*Epicautatriquetra* Werner, 1858: 15; Pinto 1991: 83.

**Distribution.** Mexico.

**Location.** Mexico: San Luis Potosí.

**Epicauta (Macrobasis) unicalcarata Champion, 1892**

*Epicautaunicalcarata* Champion, 1892: 412; Denier 1935: 160; Blackwelder 1945: 484; Pinto 1982: 406; Pinto 1991: 77.

**Distribution.** Mexico.

**Location.** Mexico: Guerrero.

**Epicauta (Macrobasis) uniforma Werner, 1944**

*Epicautauniforma* Werner, 1944: 67; 1945: 452; 1958: 5; Werner et al. 1966: 49; Selander and Agafitei 1982: 138; 1982: 201; Pinto 1991: 83.

**Distribution.** Mexico, United States.

**Location.** Mexico: Chihuahua, Durango, Sonora, Zacatecas. United States: Arizona, Colorado, New Mexico, Texas.

**Epicauta (Macrobasis) valida (LeConte, 1853)**

*Lyttasegmentata* LeConte, 1853: 342 (in part).

*Lyttavalida* LeConte, 1858: 39.

*Apterospastavalida*: LeConte 1862: 272; 1863-66: 68.

*Macrobasissegmentata*: Horn 1873: 93 (in part); Carruth 1931: 54 (in part).

*Epicautasegmenta*: Werner 1945: 490 (in part).

*Epicautavalida*: Selander & Mathieu, 1969: 118; Pinto 1991: 71.

**Distribution.** United States.

**Location.** United States: Colorado, Kansas, Louisiana, Nebraska, New Mexico, Oklahoma.

**Epicauta (Macrobasis) virgulata (LeConte, 1866)**

*Macrobasisvirgulata* LeConte, 1866: 156.

*Epicautavirgulata*: Denier 1935: 169; Werner 1945: 512 (in part); 1949: 108; Werner et al. 1966: 51; Pinto 1991: 83.

**Distribution.** Mexico, United States.

**Location.** Mexico: Baja California Norte, Baja California Sur, Sinaloa, Sonora. United States: Arizona, California.

##### Species of Epicauta (Epicauta)

**Epicauta (Epicauta) abadona Skinner, 1904**

*Epicautaabadona* Skinner, 1904: 217; Denier 1935: 152; Werner et al. 1966: 37; Adams and Selander 1979: 249; Pinto 1991: 347.

*Epicautaabadona*: Maydell 1934: 331 (*lapsus*).

*Epicautamutchleri* Maydell, 1934: 331.

**Terra typica.***E.abadona*, Holotype male, Phoenix, Arizona; ANSP. *E.mutchleri*, Holotype male, Phoenix, Arizona; AMNH.

**Distribution.** Mexico, United States.

**Location.** Mexico: Sinaloa, Sonora. United States: Arizona.

**Epicauta (Epicauta) abeona Pinto, 1980**

*Epicautaabeona* Pinto, 1980: 69; 1991: 272.

**Terra typica.** Holotype male, Ixtlán del Río, Nayarit; LACM.

**Distribution.** Mexico.

**Location.** Mexico: Aguascalientes, Distrito Federal, Jalisco, Mexico, Nayarit.

**Epicauta (Epicauta) adspersa (Klug, 1825)**

*Lyttaadspersa* Klug, 1825: 434.

*Epicautaconspersa* Curtis, 1845: 472.

*Cantharisadspersa* Burmeister, 1881: 29; Berg 1881: 307; Gemminger and Harold 1870: 2147.

*Epicautaadspersa*: Bruch 1914: 403; Borchmann 1917: 70; Denier 1935b: 152; Bosq 1943: 10; Blackwelder 1945: 482; Di Iorio 2004: 165; Campos-Soldini and Roig-Juñent 2015: 20; Pinto and Bologna 2016: 205.

**Terra typica.** one Type (s?) Mendoza, Argentina; MLPA location unknown.

**Distribution.** Argentina, Brazil, Peru, Uruguay.

**Location.** Argentina: Buenos Aires, Córdoba, Chubut, Entre Ríos, La Pampa, La Rioja, Mendoza, Neuquén, Río Negro, Salta, San Juan, Santa Fe, Santiago del Estero, Tucumán. Brazil. Peru: Junin. Uruguay: Montevideo.

**New records** (Figure 1): Argentina: Buenos Aires (Flores, La Plata, Las Martinetas, San Nicolás, Tandil); Chubut (Trelew); Córdoba (Calamuchita, El Sauce, Río IV, Salsacate, Villa María); Entre Ríos (Concordia, Diamante, Federación, Gualeguaychú, Villaguay) La Pampa (General Pico); La Rioja (Nonogasta, Villa Unión); Mendoza (Cacheuta, Los Pejecitos, Puesto la Obligación, San Rafael, Santa Rosa, Uspallata); Neuquén (Zapala); Río Negro (Choele Cheol); Santa Fe (Reconquista, Rosario); San Juan (Caligasta, Valle Fértil, Villa Nueva, Zonda). Brazil: Mato Grosso (states labeled only). Uruguay: Montevideo (Canelones, Maldonado, Minas, Peñarol, Piriapolis, Punta del Este) [MLPA, Barriga-Tuñón, IADIZA, FIMLA, MACN].

**Epicauta (Epicauta) aemula (Fischer, 1827)**

*Cantharisaemula* Fischer, 1827: 20; Gemminger and Harold 1870: 2147.

*Lyttaaemula* Hagg-Rutenberg, 1880: 53.

*Epicautaaemula*: Borchmann 1917: 70; Denier 1935: 152; Blackwelder 1945: 482; Adams and Selander 1979: 255.

**Terra typica.** Type (s?) Brazil; location unknown.

**Distribution.** Bolivia, Brazil.

**Location.** Bolivia: Coroico. Brazil: São Paulo.

**New records** (Figure 1): Brazil: Minas Gerais (Viçosa); Bello Horizonte (Minas); Rio de Janerio (Itatiaia) [MLPA].

**Epicauta (Epicauta) afoveata Werner, 1949**

*Epicautaafoveata* Werner, 1949: 103; 1955: 5; Pinto 1972: 256; 1991: 135.

**Terra typica.** Holotype male, Borrego, California; MCZC.

**Distribution.** Mexico, United States.

**Location.** Mexico: Baja California Norte, Cedros Island. United States: California.

**Epicauta (Epicauta) albolineata (Dugès, 1877)**

*Cantharisalbolineata* Dugès, 1877: 64; Vázquez and Zaragoza 1979: 579.

*Epicautaalbolineata*: Dugès 1889: 84; Champion 1892: 416; Denier 1935: 152; Blackwelder 1945: 482; Werner 1945: 451; Kaszab 1960: 285; Werner et al. 1966: 23; Pinto 1991: 114.

*Epicautaduplicata* Casey, 1891: 172.

*Epicautacostaricensis* Kaszab, 1960: 284.

**Terra typica.** Holotype male, Tupataro, Guanajuato, Mexico; UNAM.

**Distribution.** Costa Rica, Guatemala, Mexico, United States.

**Location.** Costa Rica: Belvedero, Santa Elena. Guatemala: Purulha. Mexico: Aguascaliente, Colima, Guanajuato, Guerrero, Jalisco, Mexico, Michoacán, Morelos, Nayarit, San Luis Potosí, Sonora. United States: Arizona.

**Epicauta (Epicauta) albomarginata (Mäklin, 1875)**

*Cantharisalbomarginata* Mäklin, 1875: 625.

*Epicautaalbomarginata*: Denier 1935: 152; Denier 1940: 419; Blackwelder 1945: 482.

*Epicautadenieri* Pic, 1933: 26; Denier 1935: 152.

*Epicautalimbaticollis* Pic, 1924: 2; Denier 1935: 152.

**Terra typica.** Unknown.

**Distribution.** Bolivia, Paraguay, Peru.

**Location.** Bolivia: Lagunillas, Santa Cruz. Paraguay: San Carlos, Río Apa, between Punta Apa and Vella Vista; Santa Cruz. Peru.

**New records** (Figure 1): Bolivia: Santa Cruz (departament labeled only) [MLPA].

**Epicauta (Epicauta) alphonsii Horn, 1874**

*EpicautaMaura* LeConte, 1851: 162; Horn 1873: 97; Fall 1901: 184; Moore 1937: 42.

*LyttaMaura*: LeConte 1853: 339.

*Epicautaalphonsii* Horn, 1874: 364; Denier 1935: 152; Pinto 1991: 138.

*Epicautapunticollis*: MacSwain 1943: 364.

*Epicautacalifornica* Werner, 1945: 479 (n. repl. name for *E.Maura* LeC, 1851, nec Faldeman, 1833); Werner 1955: 3; Middlekauf 1958: 9, 10; Pinto 1972: 256; Doyen and Opler 1973: 308.

**Terra typica.***E.maura* LeConte, lectotype male (designated by Werner 1945), California; MCZC. *E.alphonsii* Horn holotype female, Mariposa Co., California; MCZC.

**Distribution.** United States.

**Location.** United States: California.

**Epicauta (Epicauta) andersoni Werner, 1944**

*Epicautaandersoni* Werner, 1944: 66; 1945: 444; Vaurie 1950: 23; Werner et al. 1966: 40; Arnold 1976: 24; Pinto 1978: 57; 1980: 54; 1991: 273.

**Terra typica.** Holotype male, Gallo Springs, New Mexico; USNM.

**Distribution.** Mexico, United States.

**Location.** Mexico: Chihuahua. United States: Arizona, Colorado, Kansas, New Mexico, Oklahoma, Utah.

**Epicauta (Epicauta) anthracina (Erichson, 1848)**

*Lyttaanthracina* Erichson, 1848: 566.

*Epicautaanthracina*: Blackwelder 1945: 482; Kaszab 1960: 41; Pinto and Bologna 2016: 205.

**Terra typica.** Unknown.

**Distribution.** Brazil, Guyana, Peru.

**Location.** Brazil: Amazonas. Guayana. Peru: Coronel Portillo.

**New records** (Figure 1): Brazil: Amazonas (Manicoré, Tefé). Peru: Coronel Portillo (Pucallpa; Río Ucayali) [MLPA].

**Epicauta (Epicauta) apache Pinto, 1980**

*Epicautamaculata*: Werner 1945: 441 (in part); 1949: 96; Dillon 1952: 393 (in part); Werner et al. 1966: 38; Pinto 1975: 429; Arnold 1976: 23.

*Epicautaapache* Pinto, 1980: 62; 1991: 273.

**Terra typica.** Holotype male Willcox, Arizona; CASC.

**Distribution.** Mexico, United States.

**Location.** Mexico: Chihuahua, Sonora. United States: Arizona, Colorado, Kansas, Nebraska, Oklahoma, Texas.

**Epicauta (Epicauta) apure Adams & Selander, 1979**

*Epicautaapure* Adams & Selander, 1979: 255.

**Terra typica.** Holotype male, San Fernando, Apure, Venezuela; AMNH.

**Distribution.** Venezuela, Trinidad, Tobago.

**Location.** Venezuela: Apure, Monagas. Trinidad: George. Tobago: country labeled only.

**Epicauta (Epicauta) aragua Adams & Selander, 1979**

*Epicautagrammica*: Denier 1933: 39 (in part); 1935: 20 (in part).

*Epicautaaragua* Adams & Selander, 1979: 251.

**Terra typica.** Holotype male, Maracay, Aragua, Venezuela; AMNH.

**Distribution.** Colombia, Costa Rica, El Salvador, Honduras, Panama, Venezuela.

**Location.** Colombia: Magdalena, Meta, Santander. Costa Rica: Guanascaste, San José Higuito. El Salvador: La Libertad, San Salvador. Honduras: Copán, Francisco Morazán. Panama: Canal Zone, Chiriquí, Coclé. Venezuela: Apure, Aragua, Bolivar, Carabobo, Distrito Federal, Guarico, Miranda, Monagas, Portuguesa, Trujillo, Zulia.

**Epicauta (Epicauta) assimilis (Haag-Rutembreg, 1880)**

*Lyttaassimilis* Haag-Rutenberg, 1880: 26.

*Epicautaassimilis*: Denier 1935: 152; Blackwelder 1945: 482.

**Terra typica.** Type (s?) Rio Grande, Brazil; “Mus. Vind. and Haag-Rutenberg (in part); ZSBS.

**Distribution.** Argentina: Chaco, Córdoba, Misiones, Tucumán. Brazil. Paraguay: Alto Paraná. Uruguay: Cerro Largo.

**New records** (Figure 1): Argentina: Chaco (Charata); Córdoba (Calamuchita); Misiones (Puerto Aguirre); Tucumán (Villa Monti). Paraguay: Alto Paraná. Uruguay: Cerro Largo [MLPA, MACN].

**Epicauta (Epicauta) aspera Werner, 1944**

*Epicautaaspera* Werner, 1944: 70; 1945: 488; 1949: 105; Werner et al. 1966: 41; Arnold 1976: 24; Pinto 1991: 141.

**Terra typica.** Holotype male Salida, Colorado; MCZC.

**Distribution.** United States.

**Location.** United States: Arizona, Colorado, Kansas, New Mexico, Oklahoma, Texas.

**Epicauta (Epicauta) aterrima (Klug, 1825)**

*Lyttaaterrima* Klug, 1825: 432; Burmeister 1881: 27.

*Cantharisaterrima*: Gemminger and Harold 1870: 2148; Berg 1881: 305.

*Epicautaaterrima*: Bruch 1911: 403; Borchmann 1917: 71; Denier 1935: 152; Blackwelder 1945: 482.

**Terra typica.** Type (s?) Brazil; MLPA.

**Distribution.** Argentina, Brazil.

**Location.** Argentina: Corrientes, Puerto Cazador. Brazil: Amazonas.

**New records** (Figure 1): Argentina: Corrientes (Puerto Cazador). Brazil: Bello Horizonte (Minas); Goyas (Río Verde); Río de Janeiro (State labeled only) [MLPA].

**Epicauta (Epicauta) atomaria (Germar, 1821)**

*Lyttaatomaria* Germar, 1821: 154; Burmeister 1881: 29; Berg 1881: 307; Martínez 1992: 5.

*Lyttapunctata* Germar, 1824: 287.

*Cantharisgermari* Fischer, 1827: 24.

*Cantharisatomaria*: Gemminger and Harold 1870: 2148.

*Epicautaatomaria*: Bruch 1914: 403; Borchmann 1917: 70; Denier 1935b: 152; Bosq 1943: 10; Hayward 1942: 22; Blackwelder 1945: 482; Viana and Williner 1974: 15; Martínez 1992: 5; Di Iorio 2004: 167; Campos-Soldini 2015: 22.

**Terra typica.***Lyttaatomaria*: Type (s?) Brazil.

**Distribution.** Argentina, Bolivia, Brazil, Paraguay, Uruguay.

**Location.** Argentina: Buenos Aires, Catamarca, Córdoba, Corrientes, Entre Ríos, Formosa, Jujuy, Mendoza, Misiones, La Pampa, La Rioja, Río Negro, Santiago del Estero, Salta, San Juan, San Luis, Salta, Tucumán. Bolivia: Chiquitos, Río Negro, Santa Cruz, Santiago, Tarija. Brazil: Encruzilhada, Rio Prado, Cafapara. Paraguay: Asunción, Cordillera, Departamento de San Pedro, Guaira. Uruguay: Maldonado, Montevideo, Cerro Largo.

**Epicauta (Epicauta) atrata (Fabricius, 1775)**

*Lyttaatrata* Fabricius, 1775: 260; 1781: 329; 1801: 79.

*Meloetrichrus* Pallas, 1798: 100.

*Lyttacoracina* Illiger, 1804: 171 (unnecessary repl. name for *atrata* Fabricius, see Werner 1945: 470).

*Cantharistrichrus*: Fischer 1827: 23.

*Lyttaatrataconvolvuli* Melsheimer, 1846: 53.

*Cantharistrichrus*: Gemminger and Harold 1870: 2155.

*Lyttaconvolvuli*: Le Conte 1866: 157; Horn 1873: 97.

*Epicautaconvoluli*: LeConte 1866: 157; Horn 1873: 97.

*Cantharisconvolvuli*: Gemminger and Harold 1870: 2149.

*Epicautatrichrus*: Horn 1875: 153; Wickham 1896: 34; Blatchley 1910: 1361; Chittenden 1911: 92; Ulke 1902: 54; Sherman 1913: 246; Mutchler and Weiss 1924: 9, 18; Carruth 1931: 51

*Epicautatrichura* Wellman, 1910: 24 (unnecessary replacement name for *trichrus* Pallas; see Werner 1945: 470); Denier 1935: 159.

*Epicautapensylvanica*: Borchmann 1917: 79; Staig, 1940: 135.

*Epicautaatrata*: Werner 1945: 470; Dillon 1952: 387; Pinto 1975: 451; 1991: 294; Arnold 1976: 26; Selander 1981: 757; 1982: 812; Staines 1983: 46; Berríos-Ortíz 1985: 180; Lago and Mann 1987: 5; Pinto 1991: 294.

**Terra typica.***E.atrata* Lectotype (sex?) (designated by Werner 1945) America, GUHC. *E.trichrus*, type locality unknown. *E.convolvuli*, type locality unknown.

**Distribution.** Canada, Mexico, United States.

**Location.** Canada: Manitoba. Mexico: Nuevo León, San Luis Potosí, Tamaulipas. United States: Alabama, Arkansas, District to Columbia, Florida, Georgia, Illinois, Indiana, Iowa, Kansas, Kentucky, Louisiana, Maine, Maryland, Michigan, Mississippi, Missouri, Montana, Nebraska, New Jersey, New York, North Dakota, Ohaio, Oklahoma, Pennsylvania, Sotuh Carolina, South Dakota, Texas, Virginia, West Virginia, Wyoming.

**Epicauta (Epicauta) atropos** Pinto, 1991

*Epicautaatropos* Pinto, 1991: 299.

**Terra typica.** Holotype female, Mitchel, Nebraska; CASC.

**Distribution.** Canada, United States.

**Location.** Canada: Alberta. United States: Arizona, Colorado, Kansas, Montana, Nebraska, Oklahoma, South Dakota, Utah.

**Epicauta (Epicauta) avellanea Denier, 1834**

*Epicautaavellanea* Denier, 1934.

**Terra typica.** Holotype male, Córdoba: Valle de los Reartes. MACN.

**Distribution.** Argentina.

**Location.** Argentina: Córdoba (Valle de los Reartes).

**New records** (Figure 2): Argentina: Córdoba (Calamuchita, Valle de los Reartes) [MLPA].

**Epicauta (Epicauta) aymara Denier, 1935**

*Epicautaaymara* Denier, 1935: 152.

**Terra typica.** Holotype male, Vilaconta; MLPA. Allotype female and paratype male and female, Vilaconta; DEI.

**Distribution.** Peru: Apurímac.

**New records** (Figure 2): Peru: Apurímac (Andahuaylas) [FIMLA].

**Epicauta (Epicauta) basimaculata (Hagg-Rutenberg, 1880)**

*Lyttabasimaculata* Haag-Rutenberg, 1880: 48.

*Cantharisrufescens* Dugès, 1881: 48.

*Epicautarufescens*: Dugès 1889: 75.

*Epicautabasimaculata*: Champion 1892: 406; Blackwelder 1945: 482; Pinto 1982: 407; 1991: 240.

**Terra typica.***E.basimaculata* lectotype male (designated by Pinto 1982) Mexico; ZSMC.

**Distribution.** Guatemala, Mexico.

**Location.** Guatemala: San Marcos, Talisman. Mexico: Chiapas, Colima, Guerrero, Jalisco, Mexico, Michoacán, Morelos, Nayarit, Oaxaca, Veracruz.

**Epicauta (Epicauta) batesii Horn, 1875**

*Epicautabatesii* Horn, 1875: 153; Sherman 1913: 468; Staines 1983: 46.

*Epicautabatesi*: Mutchler and Weiss 1924: 10, 18; Denier 1935: 153; Selander 1984: 3; Pinto 1991: 302.

**Terra typica.** Lectotype female (designated by Werner 1945), Savannah, Georgia; MCZC.

**Distribution.** United States.

**Location.** United States: Florida, Georgia, New Jersey, Mississippi, North Carolina, South Carolina.

**Epicauta (Epicauta) bella Mäklin, 1875**

*Epicautabella* Mäklin, 1875: 631; Borchmann 1917: 71; Denier 1935b: 153; Blackwelder 1945: 482; Campos-Soldini 2011: 578.

*Lyttaexclamationis* Berg, 1889: 120; Bruch 1914: 405; Borchmann 1917: 94.

**Terra typica.** Type (s?) *Lyttaexclamationis*, Tandil, Argentina; Uruguay; MLPA.

**Distribution.** Argentina, Bolivia, Uruguay.

**Location.** Argentina: Buenos Aires, Tandil. Uruguay (country labeled only).

**Epicauta (Epicauta) bispinosa Werner, 1949**

*Epicautabispinosa* Werner, 1949: 95; Werner et al. 1966: 39; Pinto 1975: 435; 1980: 23, 42; 1991: 118; Arnold 1976: 27; Selander 1981: 780; Selander and Agafitei 1982: 138.

**Terra typica.** Holotype male, east of Sonoita, Arizona; MCZC.

**Distribution.** United States.

**Location.** United States: Colorado, Kansas.

**Epicauta (Epicauta) boliviensi Kaszab, 1963**

*Epicautaboliviensis* Kaszab, 1963.

**Terra typica.** Holotype male, Bolivia, Cochabamba; SUNM.

**Distribution.** Bolivia.

**Location.** Bolivia: Cochabamba.

**Epicauta (Epicauta) borchmanni Denier, 1935**

*Epicautaborchmanni* Denier, 1935: 153; Blackwelder 1945: 482.

**Terra typica.** Holotype and Allotype (sex?) Jujuy; MLPA.

**Distribution.** Argentina.

**Location.** Argentina: Jujuy.

**Epicauta (Epicauta) borgmeieri Denier, 1935**

*Epicautaborgmeieri* Denier, 1935: 153; Blackwelder 1945: 482.

**Terra typica.** Holotype male (?) Campos Grande, Mato Grosso, Brazil; MLPA.

**Distribution.** Brazil.

**Location.** Brazil: Mato Grosso, Campo Grande, Porto Murthino.

**Epicauta (Epicauta) bosqi Denier, 1935**

*Epicautabosqi* Denier, 135: 135; Bosq 1943: 11; Blackwelder 1945: 482; Viana and Williner 1974: 11; Hayward 1960: 22; Di Iorio 2004: 168; Campos-Soldini and Roig-Juñent 2011: 24.

**Terra typica.** Holotype female, Rosario, Santa Fe, Argenitna, MLPA.

**Distribution.** Argentina.

**Location.** Argentina: Buenos Aires, Chaco, Córdoba, Corrientes, Entre Ríos, Formosa, Misiones, Neuquén, Salta, Santa Fe, Santiago del Estero.

**New records** (Figure 2): Argentina: Buenos Aires (Villa Gesel); Córdoba (Baratelli, Calamuchita, Calchín); Formosa (Laguna Yema); Salta (Güemez); Santa Fe (Quebrada Bulava, La Rubia, Recreo, Rosario, Santa Fe, Santo Tomé) [IADIZA, Barriga-Tuñón, MLPA, MACN, MCNFA].

**Epicauta (Epicauta) brasilera Selander, 1981**

*Epicautabrasilera* Selander, 1981: 587.

**Terra typica.** Holotype male, São Domingo, Mato Grosso do Sul, Brazil; MNH.

**Distribution.** Brazil.

**Location.** Brazil: Goiás, Mato Grosso, Mato Grosso do Sul, São Paulo.

**Epicauta (Epicauta) bruchi Borchmann, 1930**

*Epicautabruchi* Borchmann, 1930: 93; Denier 1935b: 154; Blackwelder 1945: 482; Martínez 1992: 5; Di Iorio 2004: 168.

**Terra typica.** Type (s?) Catamarca: Santa María, Tucumán: Tafí del Valle; MACN.

**Distribution.** Argentina.

**Location.** Argentina: Catamarca, Chubut, Córdoba, Mendoza, Misiones, Neuquén, Salta, Tucumán. Perú (country labeled only).

**New records** (Figure 2): Argentina: Catamarca (La Ciénaga, Valle de Santa María); Chubut (Province labeled only); Córdoba (Capilla del Monte); Mendoza; Misiones; Neuquén; Salta (Cafayate); Tucumán (La Quebradita, Tafí del Valle) [MLPA, FIMLA, MACN].

**Epicauta (Epicauta) brunnea Werner, 1944**

*Epicautabrunnea* Werner, 1944: 67; 1945: 454; Dillon 1952: 400; Werner et al. 1966: 33; Pinto 1982: 402; 1991: 217.

*Epicautainnomina* Dillon, 1952: 401.

**Terra typica.***E.brunnea* holotype male, Alpine, Texas; MCZ.

**Distribution.** Mexico, United States.

**Location.** Mexico: Chihuaha, Sinaloa, Sonora. United States: Arizona, New Mexico, Texas.

**Epicauta (Epicauta) brunneipennis (Haag-Rutenberg, 1880)**

*Lyttabrunneipennis* Haag-Rutenberg, 1880: 29.

*Cantharisbrunneipennis*: Burmeister 1881: 24; Berg 1881: 304.

*Epicautabrunneipennis*: Bruch 1914: 403; Borchmann 1917: 72; Denier 1935b: 154, 1940: 419; Blackwelder 1945: 482; Viana and Williner 1974: 14; Di Iorio 2004: 168; Campos-Soldini and Roig-Juñent 2011: 579.

**Terra typica.** Type (s?) Buenos Aires; ZSBS.

**Distribution.** Argentina, Brazil, Paraguay, Uruguay.

**Location.** Argentina: Buenos Aires, Catamarca, Chaco, Córdoba, Corrientes, Chaco, Entre Ríos, Formosa, Mendoza, Misiones, Salta, San Luis, Tucumán. Brazil: São Paulo. Uruguay.

**New records** (Figure 2): Argentina: Entre Ríos (Chajarí, Parque Nacional el Palmar); Chaco (Resistencia); Córdoba (Salsacate); Corrientes (Ibarreta); Formosa (Bartolomé de las Casas, Parque Río Pilcomayo, Pirané); Misiones (Departamentode Concepción); Salta (Chicoana, La Viña, Orán, Sumalago); San Luis (San Gerónimo); Santa Fe (Villa Ana); Tucumán (province labeled only). Uruguay: country labeled only [MLPA, Barriga-Tuñón, FIMLA, MACN].

**Epicauta (Epicauta) bucephala Kaszab, 1960**

*Epicautabucephala* Kaszab, 1960: 406; Pinto and Bologna 2016: 205.

**Terra typica.** Holotype male and Allotype female, Andahuaylas, Peru; SMB.

**Distribution.** Peru.

**Location.** Peru: Andahuaylas, 3800 m; Apurimac.

**Epicauta (Epicauta) callosa LeConte, 1866**

*Epicautacallosa* LeConte, 1866: 158; Horn 1973: 99; Packard 1889: 225; Milliken 1921: 6; Carruth 1931: 52; Denier 1935: 154; Gilberston and Horsfall 1940: 20; Werner 1945: 468; 1949: 103; Vaurie 1950: 29; Dillon 1952: 384; Selander 1954: 24; Parker and Wakeland 1957: 26; Burke 1963: 53; Werner et al. 1966: 43; Goeden 1971; Rees 1973: 179; Arnold 1976: 25; Pinto 1977: 141; 1991: 304.

*Canthariscallosa*: Gemminger and Harold 1870: 2148.

*Epicautapseudocallosa* Dillon, 1952: 387.

**Terra typica.***E.callosa*, Lectotype female (designated by Werner 1945) Nebraska; MCZC. *E.pseudocallosa*, Holotype male, MacLennon Co., Texas; TAMU.

**Distribution.** Mexico, United States.

**Location.** Mexico: Chihuahua, Coahuila, Nuevo León, Tamaulipas. United States: Arizona, Arkansas, Colorado, Kansas, Louisiana, Montana, Nebraska, New Mexico, Oklahoma, South Dakota, Texas, Wyoming.

**Epicauta (Epicauta) candidata Champion, 1892**

*Epicautacandidata* Champion, 1892: 426; Denier 1935: 154; Blackwelder 1945: 482; Dillon 1952: 395; Pinto 1991: 120.

**Terra typica.** Holotype female, Villa Lerdo, Durango; BMNH. Pinto (1991) indicates that the type is damage.

**Distribution.** Mexico, United States.

**Location.** Mexico: Chihuahua, Coahuila, Durango, Zacatecas. United States: Texas.

**Epicauta (Epicauta) carmelita (Haag-Rutenberg, 1880)**

*Lyttacarmelita* Haag-Rutenberg, 1880: 46.

*Epicautacarmelita*: Dugès 1889: 61; Champion 1892: 417; Maydell 1934: 332; Denier 1935: 154; 1940: 419; Pinto 1982: 407; 1991: 123; Mathieu 1983: 158.

**Terra typica.** Lectotype male (designated by Pinto, 1982) “N. Granada (former name of Colombia and Panama); BMNH.

**Distribution.** Colombia, Costa Rica, Guatemala, Honduras, Mexico, Nicaragua, Panama, Venezuela.

**Location.** Costa Rica: Bebedero, Canas, La Pacifica, Santa Elena, Taboga. Guatemala: Coatepeque, Jalpatagua, San Sebastián. Honduras: Guaimaca, La Paz, Siquatepeque, Zamorano. Mexico: Chiapas, Oaxaca, Veracruz. Nicaragua: Chontales, Rivas. Panama: Canal Zone, El Coronero. Venezuela: Merida.

**New records** (Figure 2): Venezuela: Mérida (Mérida) [MLPA].

**Epicauta (Epicauta) cardui (Dugès, 1889)**

*Henons* [sic] *conferta*: Dugès 1869: 102.

*Henonscardui* Dugès, 1889: 36; Champion 1891: 368; Van Dayke 1928: 409, 411.

*Epicautacardui*: Pinto 1991: 174.

**Terra typica.** Neotype male (designated by Pinto, 1991), Toluca, Mexico.

**Distribution.** Mexico.

**Location.** Mexico: Aguascalientes, Colima, Distrito Federal, Durango, Guanajuato, Jalisco, Mexico, Michoacán, Morelos, Oaxaca, Querétaro.

**Epicauta (Epicauta) castadiva Pinto, 1991**

*Epicautacastadiva* Pinto, 1991: 176.

**Terra typica.** Holotype male, south of Valle Nacional (4200’), Oaxaca, Mexico; CASC.

**Distribution.** Mexico.

**Location.** Mexico: Oaxaca.

**Epicauta (Epicauta) caustica Rojas, 1857**

*Epicautacaustica* Rojas, 1857: 441; Denier 1935: 22; Blackwelder 1945: 482; Selander 1981: 584.

*Canthariscaustica*: Gemminger and Harold 1870: 2148.

*Lyttacaustica*: Haag-Rutenberg 1880: 53.

**Terra typica.** Neotype male (designated by Selander,1981) San Juan de las Morras, Guarico, Venezuela.

**Distribution.** Panamá, Venezuela.

**Location.** Argentina: Córdoba; Misiones. Panamá: Canal Zone. Venezuela: Apure, Aragua, Bolívar, Carabobo, Guarico, Zulia. Paraguay: Villa Florida

**New records** (Figure 3): Argentina: Córdoba (Calamuchita, San Miguel de los Ríos, Yacanto); Misiones (Pindapoy). Paraguay: Villa Florida [MLPA, MACN].

**Epicauta (Epicauta) cavernosa (Courbon, 1855)**

*Canthariscavernosa* Courbon, 1855: 1006.

*Canthariscavernosa*: Gemminger and Harold 1870: 2148.

*Canthariscavernosa*: Berg 1881: 306.

*Cantharisnigropunctata* Burmeister, 1881: 28 (partim); Berg 1881: 306; Bruch 1914: 404; Borchmann 1917: 72.

*Epicautacavernosa*: Borchmann 1917: 72; Bruch 1914: 404; Blackwelder 1945: 482; Viana and Williner 1974: 15; Campos-Soldini and Roig-Juñent 2015: 24.

**Terra typica.** Unknown.

**Distribution.** Argentina, Brazil, Uruguay.

**Location.** Argentina: Buenos Aires, Córdoba, Mendoza, San Luis. Brazil: country labeled only. Uruguay: Cerro Pelado, Cerro Largo, Cuchilla de Melo, Fraile Muerto, Punta del Este, Chuy.

**Epicauta (Epicauta) caviceps** Horn, 1873

*Epicautacaviceps* Horn, 1873: 99; 1874: 37; 1891: 43; Van Dyke 1928: 129; Maydell 1934: 329; Denier 1935: 154; Werner 1945: 481; Werner et al. 1966: 42; Pinto 1972: 256; 1991: 142.

*Epicautacaviceps*: MacSwain 1956: 59.

**Terra typica.** Lectotype male (designated by Werner, 1945) Arizona; MCZC.

**Distribution.** United States.

**Location.** United States: Arizona, California, Nevada, Utha.

**Epicauta (Epicauta) cazieri Dillon, 1952**

*Epicautacazieri* Dillon, 1952: 394; Pinto 1975: 441; 1991: 119.

**Terra typica.** Holotype male, Sierra Blanca, Texas; AMNH.

**Distribution.** United States.

**Location.** United States: Kansas, New Mexico.

**Epicauta (Epicauta) cicatrix Werner, 1951**

*Epicautacicatrix* Werner, 1951: 5; Pinto 1991: 144.

**Terra typica.** Holotype male, Presidio, Texas; USNM.

**Distribution.** United States.

**Location.** United States: Texas.

**Epicauta (Epicauta) cinctipennis (Chevrolat, 1834)**

*Lyttacinctipennis* Chevrolat, 1834: 59.

*Canthariscinctipennis*: Dugès,1869: 101; 1870: 126.

*Epicautacinctipennis*: Dugès 1889: 85; Champion 1892: 420; Denier 1935: 154; Blackwelder 1945: 483; Wirth 1956: 22; Pinto 1982: 403, 407; 1991: 164.

**Terra typica.***E.cinctipennis* Lectotype male (designated by Pinto 1982) Mineral del Zimapan, Mexico; UZMH.

**Distribution.** Mexico, United States.

**Location.** Mexico: Guanajuato, Hidalgo, Morelos, Nuevo León, Puebla, Querétaro, San Luis Potosí. United States: Arizona, New Mexico, Texas.

**Epicauta (Epicauta) cinerea (Forster, 1771)**

*Meloecinerea* Forster, 1771: 62.

*Meloecinerea*: Pallas 1798: 98.

*Meloeclematides* Woodhouse, 1800: 213.

*Epicautafissilabris* LeConte, 1850: 232; Horn 1873: 102; 1885: 111; Gibson 1912: 87; Blackwelder 1945: 483; Werner 1945: 456.

*Lyttacinerea*: LeConte 1853: 339.

*Lyttafissilabris*: LeConte 1853: 339.

*Canthariscinerea*: Gemminger and Harold 1870: 2148.

*Epicautacinerea*: Horn 1873: 101 (in part); 1875: 153; Denier 1935: 154; Sherman 1913: 246; Carruth 1931: 300; 1970: 1786; Rees 1973: 181; Arnold 1976: 21; Staines 1983: 47.

Epicauta (Epicauta) cinerea: MacSwain 1956: 48; Pinto 1991: 177.

**Terra typica.***E.cinerea*, *E.clematides* both unknown, Pinto (1991); *E.fissilabris* Holotype female, Kakabeka [Falls], Lake Superior; MCZC.

**Distribution.** Canada, United States.

**Location.** Canada: Manitoba, Ontario, Saskatchewan. United States: Alabama, Arkansas, Colorado, Connecticut, Florida, Georgia, Illinois, Iowa, Kansas, Kentucky, Louisiana, Maine, Maryland, Massachusetts, Michigan, Minnesota, Mississippi, Missouri, Montana, Nebraska, New Hampshire, New Jersey, New York, North Carolina, North Dakota, Ohio, Oklahoma, Pennsylvania, Rhode Island, South Carolina, Tennessee, Texas, Virginia, West Virginia, Wisconsin.

**Epicauta (Epicauta) circellaris Borchmann, 1942**

*Epicautacircellaris* Borchmann, 1942; Pinto and Bologna 2016: 205.

**Terra typica.** Type (s?), Peru; type locality unknown.

**Distribution.** Peru.

**Epicauta (Epicauta) clericalis (Berg, 1881)**

*Cantharisclericalis* Berg, 1881: 308.

*Epicautaclericalis*: Borchmann 1917: 73; Bruch 1914: 404; Denier 1935: 154; Blackwelder 1945: 483; Campos-Soldini and Roig-Juñent 2011: 25.

Epicautaluteolineatavar.brevebasalis Pic, 1933: 26.

Epicautaluteolineatavar.discolineata Pic, 1933: 26; Denier 1935: 154; Blackwelder 1945: 483.

**Terra typica.** Holotype male (sex?) Misiones, Argentina, MLPA.

**Distribution.** Argentina.

**Location.** Argentina: Buenos Aires, Córdoba, Chaco, Entre Ríos, Formosa, Jujuy, Mendoza, Misiones, San Juan, Santa Fe, Santiago del Estero, Tucumán.

**New records** (Figure 3): Argentina: Buenos Aires (Villa Gesel); Córdoba (El Sauce); Jujuy (Ledesma); Santa Fe (Rosario) [Barriga-Tuñón, MLPA].

**Epicauta (Epicauta) conferta (Say, 1824)**

*Meloeconfertus* Say, 1824: 281.

*Henoustechanus* Haldemann, 1952: 377.

*Henousconfertus*: LeConte 1853: 330; Riley 1877: 562; Champion 1891: 368; Van Dyke 1928: 410; Carruth 1931: 54; Gilberston and Horsfall 1940: 19; Horsfall 1942: 93; 1943: 20.

*Henoustexanus*: LeConte 1883: 167; Vaurie 1950: 25; Selander and Pinto 1967: 411; Selander and Weddler 1969: 32; Rees 1973: 183; Arnold 1976: 20; Agafitei and Selander 1980: 11; Selander 1981: 777; Selander and Agafitei 1982: 141; Berríos-Ortíz 1985: 180. Pinto1991: 210.

Epicauta (Henous) conferta: MacSwain 1956: 45.

**Terra typica.***E.conferta* Neotype female (designated by Werner 1945) Dallas, Texas; MCZC.

**Distribution.** Mexico, United States.

**Location.** Mexico: Chihuaha, Nuevo León. United States: Arkansas, Illinois, Iowa, Kansas, Missouri, Nebraska, New Mexico, Oklahoma, South Dakota, Texas.

**Epicauta (Epicauta) convergenta Kaszab, 1963**

*Epicautaconvergenta* Kaszab, 1963: 338; Pinto and Bologna 2016: 205.

**Terra typica.** Holotype male, Machupichu, Cuzco, Peru; SUNM.

**Distribution.** Peru.

**Records.** Peru: Cusco.

**Epicauta (Epicauta) cora García-París & Ruiz, 2013**

*Epicautacora* García-París & Ruiz, 2013: 59.

**Terra typica.** Holotype sex?) Nayarit, Huaynamota, Los Sabinos, Mexico; CNIN-IBUNAM.

**Distribution.** Mexico.

**Lacality records.** Mexico: Nayarit, Río Huaynamota, Los Sabinos.

**Epicauta (Epicauta) corvina (LeConte, 1858**)

*Lyttacorvina* LeConte, 1858: 21.

*Canthariscorvina*: Gemminger and Harold 1870: 2149.

*Epicautacorvina*: Horn 1873: 96, 102; 1885: 111; Dugès 1877: 66; Champion 1892: 418; Milliken 1921: 6; Cockerell and Harris 1925: 32; Carruth 1931: 52; Denier 1935: 154; Blackwelder 1945: 483; Werner 1945: 446; Vaurie 1950: 23; Dillon 1952: 389; Werner et al. 1966: 32; Pinto 1973: 968; 1977: 139; 1991: 220; Arnold 1976: 19; Pinto and Mayor 1986: 602.

**Terra typica.** Lectotype female (designated by Werner 1945) Valley of the Gila, Arizona; MCZC.

**Distribution.** Mexico, United States.

**Location.** Mexico: Chihuahua, Coahuila, Durango, Sinaloa, Sonora. United States: Arizona, Colorado, Iowa, Nebraska, New Mexico, Oklahoma, South Dakota, Texas, Wyoming.

**Epicauta (Epicauta) corybantica** Pinto, 1991

*Epicautacorybantica* Pinto, 1991: 145.

*Epicautaalphonsii*: Fall 1901: 184; Werner 1945: 480; 1955: 3; Pinto 1972: 256.

*Epicautaalphonsei*: Moore 1937: 42.

**Terra typica.** Holotye male, S. Adelanto, San Bernardino Co., California.

**Distribution.** United States.

**Location.** United States: California.

**Epicauta (Epicauta) costata (LeConte, 1854)**

*Lyttacostata* LeConte, 1854: 84; 1858: 23.

*Pleuropomphacostata*: LeConte 1862: 273; LeConte and Horn 1883: 421; Cockerell and Harris 1925: 30; Werner 1943: 31; 1945: 426; Vaurie 1950: 38; Dillon 1952: 378; Gupta 1965: 457; 1971: 14; Werner et al. 1966: 53; Goeden 1971: 47, 48; Pinto 1973: 957; 1977: 135; Arnold 1976: 30; Selander 1981: 780.

*Canthariscostata*: Gemminger and Harold 1870: 2149.

*Epicautacostata*: Pinto 1984: 381; 1991: 228; Pinto and Mayor 1986: 602.

**Terra typica.** Holotype female Frontera (Rio Grande), New Mexico: MCZC.

**Distribution.** Mexico, United States.

**Location.** Mexico: Chihuahua, Coahuila, Durango, Sonora. United States: Arizona, California, New Mexico, Oklahoma, Texas.

**Epicauta (Epicauta) costipennis Borchmann, 1930**

*Epicautacostipennis* Borchmann, 1930: 93; Denier 1935: 154; Blackwelder 1945: 483; Campos-Soldini et al. 2009: 4.

**Terra typica.** Type (s?) Troquero, Jujuy; MACN.

**Distribution.** Argentina.

**Location.** Argentina: Jujuy.

**New records** (Figure 3): Argentina: Jujuy (Torquero) [MLPA].

**Epicauta (Epicauta) crassitarsis Maydell, 1935**

*Epicautacrassitarsis* Maydell, 1935: 72; Denier 1940: 419; Werner 1945: 439; Werner et al. 1966: 38; Adams and Selander 1979: 234; Pinto 1991: 241.

**Terra typica.** Holotype male, Tempe, Arizona; USNM.

**Distribution.** Mexico, United States.

**Location.** Mexico: Sinaloa, Sonora. United States: Arizona.

**Epicauta (Epicauta) crucerea Selander, 1981**

*Epicautacrucerea* Selander, 1981: 589.

**Terra typica.** Holtype male, Chiquitos, Santa Cruz, Bolivia; MNH.

**Distribution.** Bolivia.

**Location.** Bolivia: Santa Cruz (San José de Chiquitos, 700 m).

**Epicauta (Epicauta) cupraeola (Dugès, 1869)**

*Canthariscupraeola* Dugès, 1869: 112; Horn 1885: 107; Vásquez and Zaragoza 1979: 578.

*Cantharisrufipedes* Dugès, 1870: 163.

*Canthariscinctella* Dugès, 1877: 59.

*Lyttasubvittata* Haag-Rutenberg, 1880: 47; Pinto, 1982: 47.

*Epicautainsignis* Horn, 1885: 110; Werner 1945: 437; Dillon 1952: 397; Werner et al. 1966: 37; Pinto 1977: 137.

*Epicautarufipedes*: Dugès 1889: 64; Champion 1892: 407; Denier 1935: 22; Vaurie 1950: 17; Selander 1954: 87; 1959: 210; Pinto 1982: 404.

*Epicautacinctella*: Dugès 1889: 69.

*Epicautavittula* Beauregard, 1889: 113 (n repl. name for *E.subvittata* (Haag-Rutenberg) nec Erichson, 1848).

*Epicautacupraeola*: Champion 1982: 408; Dugès 1889: 69; Denier 1935: 152; 1940: 419; Blackwelder 1945: 483; Pinto 1991: 244; García-París and Ruiz 2013.

**Terra typica.***E.cupraeola* Holotype male, “en los cerros de Guanajauto” lost. Neotype male (designated by Pinto 1991); BMNH. *E.rufipedes* Types (s) Hacienda de la Noria, Michoacán, Mexico; CASC. *E.cinctella* Type (s) states of Veracruz, Mexico, lost; Neotype male (designated by Pinto, 1991); CASC. *E.subvittata* Lectotype male (designated by Pinto 1982) Mexico; UZMH. *E.insignis* Lectotype male (designated by Werner, 1945) Arizona; MCZC.

**Distribution.** Costa Rica, El Salvador, Guatemala, Honduras, Mexico, Nicaragua, United States.

**Location.** Costa Rica: Alajuela, Bebedero, Cabo Santa Elena, La Pacifica, Las Canas, Leberia, Palo Verde, Turrialba, Turrubales. El Salvador: La Unión, Quezaltepeque, San Andrés, San Salvador. Guatemala: Guatemala, Managua, Rabinal, Sacapulas, Salama, San Gerónimo, Zacapa. Honduras: Siguatepeque, Tegucigalpa. Mexico: Aguascalientes, Campeche, Chiapas, Chihuahua, Cohauila, Colima, Distrito Federal, Durango, Guanajuato, Guerrero, Jalisco, Mexico, Michoacán, Morelos, Nayarit, Quintana, San Luis de Potosí, Sinaloa, Sonora, Veracruz, Yucatán, Zacatecas. Nicaragua: Chontales, Jinotega, Managua, Nandaime, Rivas. United States: Arizona, New Mexico.

**Epicauta (Epicauta) curticollis Borchmann, 1930**

*Epicautacurticollis* Borchmann, 1930: 92.

**Terra typica.** Type (s?) male and female, Santa Trinidad, Paraguay; type locality unknown.

**Distribution.** Argentina: Misiones. Brazil: Mina Gerais. Paraguay (country labeled only).

**New records** (Figure 3): Argentina: Misiones (Province labeled only). Brazil: Mina Gerais (Diamantina) [MLPA].

**Epicauta (Epicauta) curvicornis (Haag-Rutenberg, 1880)**

*Lyttacurvicornis* Haag-Rutenberg, 1880: 54.

*Lyttafunebris* Haag-Rutenberg, 1880: 44.

*Macrobasisantennalis* Dugès 1881: 148; 1889: 54.

*Epicautacurvicornis*: Champion 1892: 406; Dinier 1935: 154; Blackwelder 1945: 483 Pinto 1982: 407; 1984: 378; 1991: 264.

Epicauta (Epicauta) curvicornis: Werner 1973: 461.

**Terra typica.***E.curvicornis* Lectotype male (designated by Pinto 1982) Mirador, Veracruz, Mexico; BMNH.

**Distribution.** Mexico.

**Location.** Mexico: Guerrero, Michoacán, Morelos, Oaxaca, Puebla, Veracruz.

**Epicauta (Epicauta) delicata Mathieu, 1983**

*Epiautadelicata* Mathieu, 1983: 158; Pinto 1984: 381; 1991: 230.

**Terra typica.** Holotype male, Paila, Coahuila.

**Distribution.** Mexico.

**Location.** Mexico: Coahuila.

**Epicauta (Epicauta) diagramma (Burmeister, 1881)**

*Cantharisdiagramma* Burmeister, 1881: 24.

*Cantharisgriseonigra*: Berg 1881: 304 (*partim*).

*Lyttagriseonigra*: Borchmann 1917: 405 (*partim*).

*Epicautadiagramma*: Denier 1935b: 154; 1940: 419; Blackwelder 1945: 483; Campos-Soldini 2011: 580.

**Terra typica.** Type (s?) Uruguay; type locality unknown.

**Distribution.** Argentina, Uruguay.

**Location.** Argentina: Buenos Aires: Tandil; Mendoza; San Juan; San Luis. Uruguay (country labeled only).

**New records** (Figure 3): Argentina: Mendoza (Cerro Cacheuta); San Juan (Las Tumanas, Valle Fértil); San Luis (San Gerónimo) [IADIZA, Barriga-Tuñón, FIMLA, MACN].

**Epicauta (Epicauta) diana Pinto, 1991**

*Epicautadiana* Pinto, 1991: 253. García-París and Ruiz 2013.

**Terra typica.** Holotype male, Villa Unión, Sinaloa, Mexico; CASA.

**Distribution.** Mexico.

**Location.** Mexico: Oaxaca, Sinaloa.

**Epicauta (Epicauta) dilaticornis Borchmann, 1952**

*Epicautadilaticornis* Borchmann, 1952; Pinto and Bologna 2016: 205.

**Terra typica.** Holotype female, Peru, Pampa de Cangallo; type locality unknown.

**Distribution.** Peru.

**Location.** Peru: Ayacucho; Pampa de Cangallo, 3450 m; Querobamba; Tayapampa 4025 m.

**Epicauta (Epicauta) dilatipennis Pic, 1916.**

*Epicautadilatipennis* Pic, 1916: 8; Denier 1935b: 154, 1940: 419; Blackwelder 1945: 483; Campos-Soldini and Roig-Juñent 2015: 26.

**Terra typica.** Unknown.

**Distribution.** Argentina.

**Location.** Argentina: Misiones, Santiago del Estero.

**New records** (Figure 3): Argentina: Santiago del Estero (San Ignacio) [MACN].

**Epicauta (Epicauta) diversipubescens Maydell, 1934**

*Epicautadiversipubescens* Maydell, 1934: 333; Denier 1940: 420; Werner 1945: 487; 1949: 106, 108; 1954: 110; 1955: 5; Pinto 1991: 147.

**Terra typica.** Holotype male, Albuquerque, New Mexico; USNM.

**Distribution.** United States.

**Location.** United States: New Mexico, Texas.

**Epicauta (Epicauta) emarginata Champion, 1892**

*Epicautaemarginata* Champion, 1892: 426; Vaurie 1950: 30; Denier 1935: 155; Blackwelder 1945: 483; Werner 1954: 105; Werner et al. 1966: 44; Pinto 1982: 407; 1991: 307.

*Epicautacalcarata* Werner, 1944: 70; 1945: 477; Dillon 1952: 416.

**Terra typica.***E.emarginata*, Lectotype male (designated by Pinto 1982) San Isidro, Coahuila, Mexico; BMNH.

**Distribution.** Mexico, United States.

**Location.** Mexico: Chihuahua, Coahuila. United States: Arizona, New Mexico, Texas.

**Epicauta (Epicauta) ennsi Werner, 1957**

*Epicautaennsi* Werner, 1957: 97; Hoebeke 1978: 4; Pinto 1991: 308.

**Terra typica.** Holotype male, north of Rockport, Aransas Co., Texas; SEMC.

**Distribution.** United States.

**Location.** United States: Texas.

**Epicauta (Excavata) excavata (Klug, 1825)**

*Cantharisexcavata* Klug, 1825: 440; Gemminger and Harold 1870: 2150

*Cantharissulcifrons* Chevrolat, 1829: 135; Gemminger and Harold 1870: 2154

*Epicautaexcavata*: Borchmann 1917: 74; Denier 1935: 155, 1940: 420; Blackwelder 1945: 483. Martinez 1992: 5–6; Di lorio 2004: 168; Campos-Soldini and Roig-Juñent 2011: 26.

**Terra typica.** Unknown.

**Distribution.** Argentina, Brazil, Paraguay.

**Location.** Argentina: Misiones, Jujuy, Salta. Brazil: Río Grande do Soul. Chile: Huasco. Paraguay: San Pedro.

**New records** (Figure 3): Argentina: Misiones (Departamento de Concepción, Departamento de Santa María, San Ignacio); Salta (El Abisal, Coronel Moldes, Corralito, La Viña, Rosario de Lerma, Sumalago). Brazil: Matto Grosso (Rancho Grande). Chile: Huasco (Canto del Agua). Paraguay: San Pedro (Cororo) [Barriga-Tuñón, MACN].

**Epicauta (Epicauta) excavatifrons Maydell, 1934**

*Epicautaexcavatifrons* Maydell, 1934: 330; Denier 1940: 420; Werner 1945: 484; 1955: 5; Selander 1984: 3; Pinto 1991: 149.

**Terra typica.** Holotype female, Ocala, Marion Co., Florida; ANSP.

**Distribution.** United States.

**Location.** United States: Alabama, Florida, Mississippi.

**Epicauta (Epicauta) falcolarandinaGarcía-París et. al., 2016**

*Epicautafalcolarandina* García-París, Ruiz, Sánchez-Vialas & López-Estrada, 2016: 946.

**Terra typica.** Holotype female, Venezuela, Parapara; CNIN-IBUNAM.

**Distribution.** Venezuela.

**Location.** Venezuela: Falcon, Lara.

**Epicauta (Epicauta) fallax Horn, 1885**

*Epicautafallax* Horn, 1885: 111; Fall 1901: 184; Denier 1935: 155; Werner 1945: 450; 1954: 108; Ballmer 1980: 83; Pinto 1991: 332.

*Epicautaensiformis* Werner, 1944: 68.

**Terra typica.***E.fallax*, Lectotype male (designated by Pinto 1991) Owens Valley, California; MCZC.

**Distribution.** United States.

**Location.** United States: California.

**Epicauta (Epicauta) ferruginea (Say, 1824)**

*Lyttaferruginea* Say, 1824: 298; LeConte 1853: 341.

*Cantharisferruginea*: Gemminger and Harold 1870: 2150.

*Epicautaferruginea*: LeConte 1866; Horn 1873: 153; 1875: 153; 1891: 43; Champion 1892: 425; Wickham 1896: 34; Milliken 1921: 6; Carruth 1931: 51; Denier 1935: 155; Werner 1945: 465; Vaurie 1950: 28; Dillon 1952: 386; Parker and Wakeland 1957: 26; Gupta 1965: 454; Hatch 1965: 107; Werner et al. 1966: 43; Church 1967: 756; Rees 1973: 187; Arnold 1976: 25; Kumar et al. 1976: 23; Lavigne 1976: 755; Pinto 1991: 309.

**Terra typica.** Neotype female (designated by Werner 1945) Cambridge, Nebraska; MCZC.

**Distribution.** Canada, Mexico, United States.

**Location.** Tipical Race: Canada: Alberta, Manitoba, Saskatchewan. Mexico: Chihuahua. United States: Arizona, Colorado, Idaho, Kansas, Missouri, Montana, Nebraska, Nevada, New Mexico, North Dakota, Oklahoma, South Dakota, Texas, Utah, Wyoming. East Race: United State: Texas.

**Epicauta (Epicauta) flavogrisea (Burmeisteir, 1881)**

*Lyttaflavogrisea* Haag-Rutenberg, 1880: ?.

*Cantharisflavogrisea* Burmeister, 1881: 29.

*Epicautaflavogrisea*: Denier 1935: 155; Blackwelder 1945: 483;

**Terra typica.** Type (s?) Buenos Aires: Bahía Blanca; presumably in ZSBS.

**Distribution.** Argentina: Buenos Aires. Paraguay.

**New records** (Figure 4): Argentina: Buenos Aires (Sierra de la Ventana) [MACN].

**Epicauta (Epicauta) flobcina Pinto, 1991**

*Epicautaflobcina* Pinto, 1991: 182.

**Terra typica.** Holotype male, Easton, Pennsylvania; CASC.

**Distribution.** United States.

**Location.** United States: Alabama, District of Columbia, Georgia, Illinois, Iowa, Kentucky, Massachusetts, Maryland, Minnesota, Nebraska, New Hampshire, New Jersey, New York, North Carolina, Pennsylvania, Virginia, West Virginia, Wisconsin.

**Epicauta (Epicauta) floridensis** Werner, 1944

*Epicautafloridensis* Werner, 1944: 68; 1945: 458; Arnold 1976: 22; Staines 1983: 47; Selander 1984: 3; Pinto 1991: 184.

*Epicautapseudosolani*: Dillon 1952: 395 (in part).

**Terra typica.** Holotype male, Sebring, Florida; MCZC.

**Distribution.** United States.

**Location.** United States: Alabama, Florida, Illinois, Louisiana, Missippi, Missouri, New Jersey, Oklahoma, South Carolina, Texas.

**Epicauta (Epicauta) floydwerneri Martínez, 1955**

*Lyttarubriceps* Blanchard, 1843: 200.

*Epicautafloydwerneri* Martínez, 1955: 58; 1992: 6. (New name for *E.rubriceps* (Blanchard, 1843), not *E.rubriceps* (Redtenbacher, 1842)).

**Terra typica.** Type (s?) Santa Cruz, Bolivia; MNHN, location unknown.

**Distribution.** Argentina, Bolivia, Brazil, Paraguay.

**Location.** Argentina: La Rioja, Mendoza, Jujuy, Misiones, Salta. Bolivia: Santa Cruz de la Sierra, El Cidral, Estación de Experimentación Agrícola Saavedra. Brazil: Parabía. Paraguay: Colonia Nueva Italia, Departamento de Amambay.

**New records** (Figure 4): Argentina: Misiones (Concepción); Salta (Alemanía, Capiazuti, Chiloana, Coronel Moldes, La Viña, Pocitos, Rosario de Lerma, San Martín, Sumalago). Paraguay: Departamento de Amambay [Barriga-Tuñon, MACN].

**Epicauta (Epicauta) fortis Werner, 1944**

*Epicautafortis* Werner, 1944: 69; 1945: 466; Vaurie 1950: 28; Dillon 1952: 386; Werner et al. 1966: 43; Pinto 1975: 451; Arnold 1976: 25; Pinto 1991: 25.

**Terra typica.** Holotype male, Phoenix, Arizona; MCZC.

**Distribution.** Mexico, United States.

**Location.** Typical Race: Mexico: Baja California Norte, Baja California Sur, Chihuahua, Sonora. United States: Arizona, California, Kansas, Nevada, New Mexico, Oklahoma, Texas. East Texas Race: Mexico: Tamaulipas. United States: Texas.

**Epicauta (Epicauta) franciscana Denier, 1935**

*Epicautafranciscana* Denier, 1935: 155; Blackwelder 1945: 483; Campos-Soldini et al. 2009: 4.

**Terra typica.** Holotype male, Rosario, Santa Fe, Argentina; MLPA.

**Distribution.** Argentina, Uruguay.

**Location.** Argentina: Buenos Aires, Santa Fe, La Rioja. Uruguay: Cerro Largo (Departament labeled).

**New records** (Figure 4): Argentina: Santa Fe (Rosario) [MLPA].

**Epicauta (Epicauta) fuliginosa (Oliver, 1795)**

*Cantharisfuliginosa* Oliver, 1795: 14.

*Epicautafuliginosa*: Borchmann 1917: 75.

**Terra typica.** Type (s?) unspecified locality; location, possibly in the MNHN.

**Distribution.** Colombia: country labeled only.

**Epicauta (Epcauta) fulvicornis (Burmeister, 1881)**

*Cantharisfulvicornis* Burmeister, 1881: 29; Berg, 1881: 307.

*Epicautafulvicornis*: Bruch 1914: 404; Denier 1935: 155; Bosq 1943: 11; Hayward 1942: 22; Blackwelder 1945: 483; Martínez 1992: 6; Di Iorio 2004: 168; Campos-Soldini and Roig-Juñent 2011: 26.

*Epicautatestaceicornis* Pic, 1916: 8.

*Lyttafulvicornis*: Borchmann 1917: 94.

*Epicautafourcadei* Denier, 1939: 179; Blackwelder 1945: 483; Campos-Soldini et al. 2009: 4.

**Terra typica.** two Types *Cantharisfulvicornis*, Mendoza, province labeled only; MACN. Holotype (sex?) *Epicautafourcadei*, Argentina; MLPA.

1–3) is deposited at MLPA (La Plata, Argentina).

**Distribution.** Argentina, Paraguay.

**Location.** Argentina: Chaco, Corrientes, Entre Ríos, Fromosa, La Rioja, Misiones, Santa Fe, Santiago del Estero, Salta, Tucumán.

**New records** (Figure 4): Argentina: Chaco (General Vedia, Río Tapengo); Formosa (Clorinda, Las Lomitas); Misioines (Chulumani, Iguazú) [MLPA, FIMLA, MACN].

**Epicauta (Epicauta) fumosa (Germar, 1824)**

*Lyttafumosa* Germar, 1824: 173.

*Cantharisfumosa*: Gemminger and Harold 1870: 2150.

*Anomalonyxfumosus* Denier, 1935: 161.

*Anomalonychusfumosus* Sailor, 1940: 46; Kaszab 1963: 340.

*Epicautafumosa*: Borchmann 1917: 75; Blackwelder 1945: 483; Pinto 1984: 378.

**Terra typica.***Anomalonychusfumosus* Holotype male, Alotype female and one Paratype, Santa Catharina, Brazil in the Sammlung des Museums G. Frey. *Anomalonyxfumosus*; MLPA location unknown.

**Distribution.** Argentina, Brazil.

**Location.** Argentina: Misiones. Brazil: Goyaz.

**New records** (Figure 4): Argentina: Misiones (Province labeled only). Brazil: Goyaz [MACN].

**Epicauta (Epicauta) funebris Horn, 1873**

*Epicautafunebris* Horn, 1873: 102; Milliken 1921: 6; Denier 1935: 23; Horsfall 1943: 41; Werner 1945: 447; Dillon 1952: 389; Selander and Weddle 1969: 37; Rees 1973: 188; Arnold 1976: 20; Selander 1981: 777; Pinto 1977: 141; 1991: 186.

*Epicautacinerea*: Riley 1877: 551, 561; Chittenden 1903: 24; Milliken 1921: 6; Rees 1973: 181

*Epicautapestifera* Werner, 1949: 100 (n, repl. name for *E.solani* Werner, 1945 nec Denier, 1940); Gupta 1965: 455; Selander and Weddler 1969: 37; Arnold 1976: 21; Adams and Selander 1979: 173, 217; Selander 1981: 777; 1982: 427; 1984: 3; Staines 1983: 48; Lago and Mann 1987: 5; MacCormik and Carrel 1987: 310; Blodgett and Higging 1988: 1461

Epicauta (Epicauta) solani: MacSwain 1956: 46.

**Terra typica.***E.funebris* Lectotype (designated by Werner 1945) Texas; MCZC. *E.pestifera* Holotype male, Norwood Pennsylvania; MCZC.

**Distribution.** United States.

**Location.** United States: Alabama, Arkansas, Connecticut, Delaware, District of Columbia, Florida, Georgia, Illinois, Indiana, Louisiana, Maryland, Massachusetts, Michigan, Minnesota, Mississippi, Missouri, Nebraska, New Hampshire, New Jersey, New York, North Carolina, Ohio, Oklahoma, Pennsylvania, Rhode Island, South Carolina, South Dakota, Tennessee, Texas, Virginia, West Virginia, Wisconsin.

**Epicauta (Epicauta) geniculata (Haag-Rutenberg, 1880)**

*Lyttageniculata* Haag-Rutenberg, 1880: 28.

*Epicautageniculata*: Blackwelder 1945: 483.

**Terra typica.** Type (?s) Brazil, presumably in ZSBS.

**Distribution.** Argentina, Brazil, countries labeled only.

**Epicauta (Epicauta) grammica (Fischer, 1827)**

*Cantharisgrammica* Fischer, 1827: 19; Gemminger and Harold 1870: 2151.

*Epicautagrammica*: Borchmann 1917: 75; Denier 1935: 155, 1940: 420; Blackwelder 1945: 483; Martínez 1992: 6; Campos-Soldini and Roig-Juñent 2011: 27.

*Epicautafidelis* Brethes, 1925: 14.

**Terra typica.***Cantharisgrammica*, Type (s?) Brazil; RMM, present location unknown. *E.fidelis* Holotype (?) Santa Fe, Argentina; MACN.

**Distribution.** Argentina, Bolivia, Brazil, Colombia, Costa Rica, El Salvador, Guatemala, Honduras, Panama, Paraguay, Uruguay and Venezuela.

**Location.** Argentina: Chaco, Córdoba, Corrientes, Entre Ríos, Formosa, Jujuy, Misiones, Salta, Santa Fe, Rio Negro, Santa Fe, Salta, Tucumán. Paraguay: Departamento de San Pedro. Bolivia: Cochabamba. Brazil: Mato Grosso, Minas Gerais, São Paulo. Colombia: Magdalena, Meta, Santander, Vélez, Guanacaste, Puntarenas, San José, Coyolar. El Salvador: La Libertad. Guatemala: Izabal, Cuyotenango. Honduras: Copán, Francisco Morazón. Panama: Canal Zone, Coclé, Panamá. Uruguay: Rio Negro. Venezuela: Apure, Aragua, Bolívar, Carabobo, Distrito Federal, Miranda, Monagas, Portuguesa, Sucre, Trujillo, Zulia.

**New records** (Figure 4): Argentina: Chaco (Resistencia, Saenz Peña); Corrientes (Santo Tomé); Entre Ríos (Concordia, Parque Nacional el Palmar); Formosa (Campos Villafañe, Río Pilcomayo); Jujuy (Ledesma); Misiones (Iguazú, Piñalito, Santa María); Salta (Cafayate, Las Lajitas, Paso del Rey, Sumalao); Santa Fe (Piquete). Brazil: Mato Grosso (Rancho Grande) [Barriga-Tuñon, MLPA, MACN].

**Epicauta (Epicauta) griseonigra (Fairmaire, 1873)**

*Cantharisgriseonigra* Fairmaire, 1873: 73; Berg 1881: 304 (partim).

*Canthariscentralis* Burmeister, 1881: 25.

*Lyttagriseonigra*: Bruch 1914: 405 (partim).

Epicautacentralisvar.ochraceocincta Pic, 1916: 22

*Epicautagriseonigra*: Borchmann 1917: 76 (partim); Denier 1935b: 156 (partim); Blackwelder 1945: 483 (partim); Viana and Williner 1974: 15; Martínez 1992: 6–7; Campos-Soldini 2011: 581.

*Epicautacentralis*: Borchmann 1917: 72; Bruch 1914: 404; Blackwelder 1945: 483; Di Iorio 2004: 168.

**Terra typica.***Canthariscentralis*, Type (s?) Córdoba, La Rioja, Santiago del Estero; MHNP.

**Distribution.** Argentina, Uruguay.

**Location.** Argentina: Catamarca, Córdoba, Entre Ríos, Formosa, La Rioja, Mendoza, Salta, San Juan, San Luis, Santiago del Estero, Tucumán. Uruguay: country labeled only.

**New records** (Figure 5): Argentina: Córdoba (Calamuchita, El Sauce, Mansilla); Formosa (Bartolomé de las Casas, El Colorado, Laguna Yema); Salta (Chioacán, Salta Forestal); San Juan (Las Tunas); San Luis (El Balde, San Gerónimo) [Barriga Tuñón].

**Epicauta (Epicauta) heterodera Horn, 1891**

*Epicautaheterodera* Horn, 1891: 43; Maydell 1934: 333; Denier 1935: 156; Werner 1945: 478; Selander 1984: 3; Pinto 1991: 315.

*Epicautawatsoni* Blatchley, 1918: 58.

**Terra typica.***E.heterodera* Lectotype female (designated by Werner 1945) northern Florida; MCZC. *E.watsoni*, Holotype female, near Gainsville, Florida; PURC.

**Distribution.** United States.

**Location.** United States: Alabama, Florida, Georgia, Louisiana, Mississippi, North Carolina.

**Epicauta (Epicauta) hieroglyphica (Haag-Rutenberg, 1880)**

*Lyttahieroglyphica* Haag-Rutenberg, 1880: 26.

*Epicautahieroglyphica*: Borchmann 1917: 76; Blackwelder 1945: 483.

**Terra typica.** Type (s?) Brazil; presumably in ZSBS.

**Distribution.** Colombia and Brazil, countries labeled only.

**New records** (Figure 5): Colombia: Barranquilla (Distrit labeled only) [MLPA].

**Epicauta (Epicauta) horni Champion, 1892**

*Canthariscinerea* Dugès, 1869: 160.

*Cantharisvicina* Dugès, 1881: 147 (inappropriate repl. name). Horn 1885: 107.

*Epicautavicina*: Dugès 1889: 70.

*Epicautahorni*: Champion 1892: 412 (n. repl. name of *E.cinereas* (Dugès), nec Foster, 1771; and for *E.vicina* (Dugès), nec Haag-Rutenberg, 1880); Denier 1935: 156; Blackwelder 1945: 483; Pinto 1991: 274.

**Terra typica.** Neotype male (designated by Pinto 1991) Guanajuato, Mexico; BMNH.

**Distribution.** Mexico.

**Location.** Mexico: Colima, Guanajuato, Tamaulipas.

**Epicauta (Epicauta) hubbelli Werner, 1973**

Epicauta (Epicauta) hubbelli Werner, 1973: 460.

*Epicautahubbelli*: Pinto 1984: 378; 1991: 266.

**Terra typica.** Holotype male, Mexico, Chaipas; MCZC.

**Distribution.** Mexico.

**Location.** Mexico: Chiapas; Oaxaca.

**Epicauta (Epicauta) imitatrix Kaszab, 1960**

*Epicautaimitatrix* Kaszab, 1960: 405; Pinto and Bologna 2016: 205.

**Terra typica.** Holotype male and Allotype female, Puna de Andahuaylas, 4000 m, Peru; SMB.

**Distribution.** Peru.

**Location.** Peru: Apurímac, Ayacucho, Huanta, 2660 m.

**Epicauta (Epicauta) impressifrons Van Dayke, 1928**

*Epicautaimpressifrons* Van Dayke, 1928: 262; 1929: 129; Denier 1935: 156; Werner 1945: 482; 1945: 482; 1949: 103, 105; 1955: 5; Pinto 1972: 256; 1991: 150.

**Terra typica.** Holotype male, Plam Springs, Riverside Co., California; CASC.

**Distribution.** United States.

**Location.** United States: California.

**Epicauta (Epicauta) inconstants (Fischer, 1827)**

*Cantharisinconstants* Fischer, 1827: 17; Gemminger and Harold 1870: 2151.

*Epicautainconstants*: Borchmann 1917: 76; Denier 1935: 156; 1940: 420; Blackwelder 1945: 483.

**Terra typica.** Unknown.

**Distribution.** Paraguay, Brazil.

**Location.** Paraguay: Villeta. Brazil (Country labeled only).

**New records** (Figure 5): Paraguay: Villeta (Colonia Nueva Italia) [MLPA].

**Epicauta (Epicauta) insueta Werner, 1955**

*Epicautainsueta* Werner, 1955: 11; Pinto 1991: 151.

**Terra typica.** Holotype male, Toluca, Mexico; AMNH.

**Distribution.** Mexico.

**Location.** Mexico: Toluaca.

**Epicauta (Epicauta) jeffersi Pinto, 1980**

*Epicautanormalis*: Werner, 1949: 96 (in part); Werner et al. 1966: 38 (in part); Arnold 1976: 23.

*Epicautajeffersi* Pinto, 1980: 56; 1991: 275.

**Terra typica.** Holotype male, southeast of Willcox, Arizona; CASC.

**Distribution.** United States.

**Location.** United States: Arizona, Colorado, Oklahoma.

**Epicauta (Epicauta) jimenezi Dugès, 1889**

*Epicautajimenezi* Dugès, 1889: 73; Champion 1892: 417; Denier 1935: 156; Vaurie 1950: 23; Blackwelder 1945: 483; Werner 1954: 110; Werner et al. 1966: 32; Vázquez and Zaragoza 1979: 580; Pinto 1991: 222.

*Epicautanigropilosa* Maydell, 1934: 332; Denier 1940: 421.

**Terra typica.***E.jimenezi* Holotype (sex?) Guadalajara, Mexico; UNAM. *E.nigropilosa* Holotype (sex?) Guadalajara, Mexico; UNAM.

**Distribution.** Mexico, United States.

**Location.** Mexico: Durango, Jalisco, Sinaloa, Sonora. United States: Arizona.

**Epicauta (Epicauta) kansanas Werner, 1944**

*Epicautakansanas* Werner, 1944: 70; 1945: 476; Ballamer 1980: 84; Pinto 1991: 332.

**Terra typica.** Holotype male, type locale unknown, erroneously labeled “Sedgewick Co., Kansas”; USNM.

**Distribution.** United States.

**Location.** United States: Kansas.

**Epicauta (Epicauta) koehleri Denier, 1940**

*Epicautakoehleri* Denier, 1940: 420; Bosq 1943: 11; Viana and Williner 1974: 11; Di Iorio 2004: 168; Campos-Soldini and Roig-Juñent 2015: 28.

**Terra typica.** Holotye male, Allotype female, Santa Fe: Sancti Spiritu; MLPA, location unknown.

**Distribution.** Argentina, Bolivia.

**Location.** Argentina: Buenos Aires, Chubut Mendoza, Golfo San Jorge, Neuquén, San Juan, Santa Cruz, Santa Fe, Río Negro. Bolivia: Nor Yungas, La Paz.

**New records** (Figure 5): Argentina: Formosa (Bermejo, Laguna Yema); Neuquén (Lago Verde, San Martín de los Andes, Río Agrio) [IADIZA, FIMLA, MACN].

**Epicauta (Epicauta) koehlerivar.solani Denier, 1940**

Epicautakoehlerivar.solani Denier, 1940: 421.

**Terra typica.** Holotype and Allotype (sex?), Argentina; MLPA, location unknown.

**Distribution.** Argentina.

**Location.** Argentina: Mendoza, Neuquén, San Juan, Santa Cruz.

**Epicauta (Epicauta) korytkowskii Kaszab, 1978**

*Epicautakorytkowskii* Kaszab, 1978: 331; Pinto and Bologna 2016: 205.

**Terra typica.** Type (s?) Santa Cruz, Peru; type locality unknown.

**Distribution.** Peru.

**Location.** Peru: Cajamarca; Santa Cruz.

**Epicauta (Epicauta) kraatzi (Haag-Rutenberg, 1880)**

*Lyttakraatzi* Haag-Rutenberg, 1880: 22.

*Epicautakraatzi*: Borchmann 1917: 76.

**Terra typica.** Type (s?) Brazil; presumably in ZSBS.

**Distribution.** Brazil (Country labeled only).

**Epicauta (Epicauta) kraussi (Haag-Rutenberg, 1880)**

*Lyttakraussi* Haag-Rutenberg, 1880: 25.

*Epicautakraussi*: Borchmann 1917: 97.

**Terra typica.** Type (s?) Irisanga, Brazil, in the collection of the “Mus. Vind. and Haag-Rutenberg (in part); ZSBS.

**Distribution.** Argentina: Córdoba. Brazil: Goyaz (Río Verde).

**New Records** (Figure 5): Argentina: Córdoba (Calamuchita) [MLPA].

**Epicauta (Epicauta) laevicornis Werner, 1973**

Epicauta (Epicauta) laevicornis Werner, 1973: 458.

*Epicautalaevicornis*: Pinto 1984: 378; 1991: 267.

**Terra typica.** Holotyopes male, Mountains and canyons north of Ajijie, Jalisco, Mexico; MCZC.

**Distribution.** Mexico.

**Location.** Mexico: Colima, Guerrero, Jalisco, Mexico, Michoacán.

**Epicauta (Epicauta) latitarsis (Haag-Rutenberg, 1880)**

*Lyttalatitarsis* Haag-Rutenberg, 1880: 33.

*Epicautalatitarsis*: Blackwelder 1945: 483; Pinto and Selander 2016: 205.

**Terra typica.** Type (s?) Peru, Coll: Haag-Rutenberg; presumably in ZSBS.

**Distribution.** Peru.

**Location.** Peru: Ayacucho.

**Epicauta (Epicauta) leopardina (Hagg-Rutenberg, 1880)**

*Lyttaleopardina* Haag-Rutenberg, 1880: 30.

*Cantharisleopardina*: Burmeister 1881: 24; Berg, 1881: 304.

*Epicautaleopardina*: Bruch 1914: 404; Borchmann 1917: 77; Denier 1935: 156; Bosq 1934: 327; 1942: 11; Blackewelder 1945: 483; Viana and Williner 1974: 11; Martinez 1992: 7; Di Iorio 2004: 169; Campos-Soldini and Roig-Juñent 2015: 27.

**Terra typica.** Syntipes (sex?) Córdoba, Argentina; in the Haag-Rutenberg Collection, ZSBM.

**Distribution.** Argentina, Brazil, Colombia.

**Location.** Argentina: Buenos Aires, Catamarca, Córdoba, Chaco, Entre Ríos, Formosa, Mendoza, Misiones, Neuquén, Salta, San Juan, San Luis, Santa Fe, Santiago del Estreo, Tucumán. Brazil: Goyaz, Río de Janeiro. Colombia: Atlántico.

**New records** (Figure 5): Argentina: Catamarca (Andalgalá); Córdoba (Agua de Oro, Calamuchita, Cruz del Eje, Ischilín Quilino, La Falda, Laboulage, La Falda); La Rioja (Talamuyuna); Formosa (Laguna Yema, Palmar Largo); Mendoza (Desaguadero, Lavalle, Ñacuñan); Salta (Alemanía, Carapari, Figueroa, María Juana, Piquete); San Juan (Las Rumanas, Valle Féritl); San Luis (Balde, San Gerónimo); Santa Fe (Arrufo, Colastiné Sur, La Rubia, Recreo, Rincón Norte, San Cristobal); Tucumán (Gobernador Garmendia) [IADIZA, FIMLA, MACN, MLPA, MCNFA].

**Epicauta (Epicauta) leucocoma Champion, 1892**

*Epicautaleucocoma* Champion, 1892: 425; Denier 1935: 156; Pinto 1982: 407; 1991: 254.

**Terra typica.** Lectotype male (designated by Pinto 1982) Tepanistlahuaca (= Tepanixtlahuaca) Oaxaca, Mexico.

**Distribution.** Mexico.

**Location.** Mexico: Guerrero, Jalisco, Oaxaca.

**Epicauta (Epicauta) limbaticollis Pic, 1924**

*Epicautalimbaticollis* Pic, 1924: 32; Pinto and Bologna 2016: 205.

**Terra typica.** Type (s?) Peru; type locality unknown.

**Distribution.** Peru: country labeled only.

**Epicauta (Epicauta) lizeri Denier, 1934**

*Epicautalizeri* Denier, 1934: 271; Denier 1940: 421; Blackwelder 1945: 483; Campos-Soldini et al. 2009: 5; Campos-Soldini and Roig Juñent 2015: 29.

**Terra typica.** Holotype, allotype, and eight paratypes (sex?), Argentina: MLPA; two paratypes, location unknown; MACN.

**Distribution.** Argentina, Bolivia.

**Location.** Argentina: Catamarca, Chaco, Jujuy, La Rioja, Salta, Santa Cruz, Santiago del Estero, Tucumán. Bolivia: Santa Cruz.

**New records** (Figure 6): Argentina: Salta (province labeled only); Tucumán (Tafí del Valle). Bolivia: Santa Cruz (Lagunilla); [MLPA, FIMLA, MACN, MLPA].

**Epicauta (Epicauta) luctifera (Fairmaire, 1873)**

*Cantharisluctifera* Fairmaire, 1873: 534; Berg 1881: 303.

*Cantharisleucoloma* Burmeister, 1881: 22.

*Lyttaluctifera*: Bruch 1914: 405.

*Epicautaluctifera*: Borchmann 1917: 77; Bosq 1943: 11; Hayward 1942: 23; Blackwelder 1945: 483; Viana and Williner 1974: 14; Di Iorio 2004: 169; Campos-Soldini 2011: 582.

**Terra typica.***Cantharisleucocoma*, Type (s?) Uruguay; type locality unknown.

**Distribution.** Argentina, Uruguay.

**Location.** Argentina: Buenos Aires; Córdoba, San Luís, Tucumán. Uruguay (Montevideo).

**New records** (Figure 6): Argentina: Córdoba (Calamuchita, El Sauce). Uruguay: Florida (Arroyo Chico); Montevideo: (Minas) [Barriga-Tuñón, MLPA].

**Epicauta (Epicauta) luteolineata Pic, 1933**

*Epicautaluteolineata* Pic, 1933: 25.

Epicautamissionumvarluteolineata: Denier 1935: 157; 1940: 421; Blackwelder 1945: 483; Di Iorio 2004: 169; Campos-Soldini and Roig-Juñent 2011: 38.

**Terra typica.** Syntypes (sex?), “Río Salado” Argentina; type locality unknown.

**Distribution.** Argentina.

**Location.** Argentina: Mendoza, Misiones, Santa Fe; Salta, Santiago del Estero, Tucumán.

**Epicauta (Epicauta) maculata (Say, 1823)**

*Lyttamaculata* Say, 1823: 298; LeConte 1853: 340.

*Lyttaconspersa* LeConte, 1853: 340; 1866: 158.

*Cantharismaculata*: Gemminger and Harold 1870: 2151.

*Epicautamaculata*: LeConte 1866: 158; Denier 1935;156; Gilberston and Horsfall 1940: 14; Larson 1943: 480; Werner 1944: 65; 1945: 441; 1949: 95 (in part); Vauri 1950: 19; Dillon 1952: 393; (in part); Arnold 1976: 23; Pinto 1980: 64; 1991: 275; Bouseman 1986: 366.

*Cantharispunctata*: Dugès 1870: 161.

*Epicautamedia* Dugès, 1889: 82.

*Epicautaconspersa*: Champion 1892: 413.

*Epicautanogales* Werner, 1944: 65; 1945: 442; 1949: 96; Dillon 1952: 391; Werner et al. 1966: 39; Pinto 1975: 430.

**Terra typica.***E.maculata*: Neotype male (designated by Werner 1945) Indianola, Nebraska; MCZC. *E.conspersa* Lectotype male (designated by Werner 1945) Missoury. *E.punctata* apparently lost. *E.media* Holotype male, Nogales, Arizona; MCZC.

**Distribution.** Canada, Guatemala, Mexico, United States.

**Location.** Canada: Manitoba, Saskatchewan. Guatemala: Chichicastenango, Huehuetenango. Mexico: Aguascalientes, Chihuahua, Coahuila, Distrito Federal, Durango, Guanajuato, Hidalgo, Jalisco, Michoacan, Nuevo León, Oaxaca, Queréteros, Sonora, Zacatecas. United States: Arizona, Colorado, Illinois, Iowa, Kansas, Montana, Nebraska, New Mexico, North Dakota, Oklahoma, South Dakota, Texas.

**Epicauta (Epicauta) magnomaculata Martin, 1932**

*Epicautamagnomaculata* Martin, 1932: 169; Maehler 1939: 65; Denier 1940: 421; Werner 1945: 445; Pinto 1980: 46; 1991: 276.

**Terra typica.** Holotype male, Ballart, Inyo Co., California; CASC.

**Distribution.** United States.

**Location.** United States: California.

**Epicauta (Epicauta) major Pic, 1924**

*Cantharismarginata*: Dugès 1877: 59.

*Epicautamarginata*: Dugès 1889: 78.

*Epicautacinerea*: Champion 1892: 421.

*Epicautacinctipennismajor* Pic, 1924: 2.

*Epicautasubatra*: Denier 1935: 22.

*Epicautamajor*: Pinto 1991: 191.

**Terra typica.** Syntypes from southern Tamaulipas and San Luis Potosí south; MNHP.

**Distribution.** Costa Rica, Guatemala, Honduras, Mexico, Panama.

**Location.** Costa Rica: Carillo, Cartago, Dos Ríos, Hamburg Farm, Juan Viñas, La Estrella, San Carlos. Guatemala: Senahu, Panzos, Tikal. Honduras: Tela. Mexico: Campeche, Chiapas, Oaxaca, Quintana Roo, San Luis Potosí, Tabasco, Veracruz, Yucatan. Panama: Cerro Salud, Maje Island.

**Epicauta (Epicauta) mexicana (Dugès, 1889)**

*Henousmexicanus* Dugès, 1889: 37; Champion, 1892: 369; Van Dyke 1928: 409.

*Epicautamexicana* Pinto, 1991: 196.

**Terra typica.** Lectotype male (designated by Pinto 1991) Mexico; HMNH.

**Distribution.** Mexico.

**Location.** Mexico: Hidalgo, Veracruz.

**Epicauta (Epicauta) minutepunctata Borchmann, 1930**

*Epicautaminutepunctata* Borchmann, 1930: 94; Denier 1935: 157; Blackwelder 1945: 483; Campos-Soldini and Roig-Juñent 2015: 31.

*Epicautarosilloi* Martínez, 1952: 255; Campos-Soldini and Roig-Juñent 2015.

**Terra typica.** Type (s?) Mendoza: Pedregal; MACN.

**Distribution.** Argentina.

**Location.** Argentina: Buenos Aires, Jujuy, Salta, San Luis: San Francisco, Tucumán.

**New records** (Figure 6): Argentina: Buenos Aires (province labeled only); Entre Ríos (Concordia); Salta (Chicoana, Salta Forestal, Tabillar) San Luis (San Framcisco); Tucumán (Río Mediano) [MLPA, Barriga-Tuñón, FIMLA, MACN].

**Epicauta (Epicauta) mirabilis Kaszab, 1963**

*Epicautamirabilis* Kaszab, 1963: 338; Pinto and Bologna 2016: 205.

**Terra typica.** Holotype male, Ayacucho, Peru; SUNM.

**Distribution.** Peru.

**Location.** Peru: Ayacucho.

**Epicauta (Epicauta) missionum (Berg, 1881)**

*Cantharismissionum* Berg, 1881: 306.

*Cantharisclericalis* Berg, 1881: 308

*Epicautamissionum* Borchmann, 1971: 78; Denier 1935b: 157; Blackwelder 1945: 483; Campos-Soldini et al. 2009: 5; Campos-Soldini and Roig-Juñent 2015: 38.

Epicautaluteolineatavar.brevebasalis Pic, 1933: 26.

Epicautaluteolineatavar.discolineata Pic, 1933: 26; Denier 1935: 154; Blackwelder, 1945: 483.

Epicautamissionumvar.luteolineata: Denier 1935b: 157; 1940: 421; Blackwelder 1945: 483; Di lorio 2004: 169.

Epicautaclericalisvar.discolineata: Denier 1935: 154; Blackwelder 1945: 483.

**Terra typica.** Holotype female, Misiones, Argentina; MLPA.

**Distribution.** Argentina, Brazil, Uruguay.

**Location.** Argentina: Buenos Aires, Córdoba, Entre Ríos, Formosa, Jujuy, La Rioja, Mendoza, Misiones, Neuquén, Rosario, Salta, San Juan, San Luis, Santa Fe, Santiago del Estero, San Luis, Tucumán. Brazil: Matto Grosso. Paraguay: San Pedro de Cororo. Uruguay: Rivera.

**New records** (Figure 6): Argentina: San Luis (Las Lajitas, San Gerónimo); Misiones (Lagunilla). Brazil: Matto Grosso (Rancho Grande). Paraguay: San Pedro (Cororo). Uruguay: Rivera (Sierra de la Rivera) [Barriga-Tuñon].

**Epicauta (Epicauta) mixta Dugès, 1889**

*Lyttaneglecta* Haag-Rutenberg, 1880: 54.

*Epicautamixta* Dugès, 1889: 83; Vázquez and Zaragoza 1979: 580; Pinto 1982: 404; 1991: 224; Pinto and Mayor 1986: 602.

*Epicautaneglecta*: Champion 1982 (in major part).

**Terra typica.** Holotype (?) Oaxaca, Mexico; UNAM.

**Distribution.** Mexico.

**Location.** Mexico: Chiapas, Distrito Federal, Guerrero, Hidalgo, Mexico, Michoacán, Morelos, Oaxaca, Veracruz.

**Epicauta (Epicauta) modesta (Haag-Rutenberg, 1880)**

*Lyttamodesta* Haag-Rutenberg, 1880: 53.

*Epicautamodesta*: Champion 1892: 423; Denier 1935: 157; Blackwelder 1945: 483; Pinto 1991: 125.

**Terra typica.** Holotype male, Mexico; ZSMC.

**Distribution.** Mexico, country labeled only.

**Epicauta (Epicauta) monachica (Berg, 1883)**

*Lyttamonachica* Berg, 1883: 68.

*Epicautamonachica*: Blanchard 1891: 495; Bruch 1914: 404; Borchmann 1917: 78; Denier 1935b: 157; Bosq 1934: 327; 1943: 11; Hayward 1942: 23; Blackwelder 1945: 483; Viana and Williner 1974: 87; Martinez 1992: 7; Di Iorio 2004: 170; Campos-Soldini et al. 2009: 5; Campos-Soldini and Roig-Juñent 2011: 39.

**Terra typica.** Lectotype female (designated by Adams and Selander 1979) Rodeo del Medio, Mendoza, Argentina; MLPA.

**Distribution.** Argentina, Bolivia, Brazil.

**Location.** Argentina: Catamarca; Córdoba, Chaco, La Rioja: Chilecito, Quebrada de Olta, Santiago del Estero, Salta, Santa Fe, Catamarca, Córdoba, Corrientes, Chaco, Formosa, La Rioja, Mendoza, Misiones, Salta, San Juan, Santa Fe, Santiago del Estero, Tucumán. Bolivia: Lagunilla. Brazil: Mato Grosso.

**Epicauta (Epicauta) monrosi Kaszab, 1960**

*Epicautamonrosi* Kaszab, 1960: 402; Pinto and Bologna 2016: 205.

**Terra typica.** Holotype and Paratype male, Cuzco, 3200–4200 m, Peru; SUMB.

**Distribution.** Peru.

**Location.** Peru: Cusco.

**Epicauta (Epicauta) montara Ballmer, 1980**

*Epicautamontara* Ballmer, 1980: 85; Pinto 1991: 333.

**Terra typica.** Holotype male, Montara, San Mateo Co., California; CASC.

**Distribution.** United States.

**Location.** United States: California.

**Epicauta (Epicauta) montei Denier, 1935**

*Epicautamontei* Denier, 1935: 157; Blackwelder 1945: 483; DiIorio 2004: 170; Campos-Soldini et al. 2009: 5.

**Terra typica.** Holotype (sex?), Allotype (sex?) and Paratype (s?) Brazil: Mina Gerais, Bello Horizonte; Goyaz, Río Verde; Río de Janeiro, Mendes; Río Grande do Soul. Uruguay: Cerro Largo; in the Sección de Entomología del Instituto de Biología Vegetal de Río de Janeiro.

**Distribution.** Brazil, Uruguay.

**Location.** Argentina: Misiones. Brazil: Bello Horizonte (Mina Gerais); Goyaz: Rio Verde; Rio de Janeiro: Mendes; Rio Grande do Soul. Uruguay: Cerro Largo.

**New records** (Figure 6): Argentina: Misiones. Brazil: Goyaz: Rio Verde; Mina Gerais (Passa quatro) [MLPA].

**Epicauta (Epicauta) nattereri Haag-Rutenberg, 1880**

*Epicautanattereri* Haag-Rutenberg, 1880: 78; Denier 1935: 157; Blackwelder 1945: 483.

**Terra typica.** Type (s?) Irizanga, Brazil; “Mus. Vind. and Haag-Rutenberg; ZSBS.

**Distribution.** Brazil.

**Location.** Brazil: Goyaz.

**New records** (Figure 6): Brazil: Goyaz (Río Verde).

**Epicauta (Epicauta) nigrans Mäklin, 1875**

*Epicautanigrans* Mäklin, 1875; Pinto and Bologna 2016: 205.

**Terra typica.** Type (s?) Peru; type locality unknown.

**Distribution.** Peru (country labeled only).

**Epicauta (Epicauta) nigropunctata (Blanchard, 1843)**

*Cantharisnigropunctata* Blanchard, 1843: 200; Gemminger and Harold 1870: 2152.

*Cantharisnigropunctata*: Burmeister 1881: 28 (partim).

*Epicautanigropunctata*: Borchmann 1917: 79; Denier 1935b: 158; Blackwelder 1945: 483; Bosq 1942: 11; Di Iorio 2004: 170; Campos-Soldini and Roig-Juñent 2015: 32.

*Epicautabreyeri* Denier, 1934: 273; 1935b: 154; Blackwelder 1945: 482.

**Terra typica.***E.breyeri* Holotype male, Allotype female, La Rioja: Patquia; MACN; MLPA location unknown.

**Distribution.** Argentina, Bolivia, Brazil, Uruguay.

**Location.** Argentina: Catamarca, Chaco, Córdoba, Corrientes, Misiones, Mendoza, Salta. Bolivia Chulumaní, Coroico. Brazil: Parana. Uruguay: Montevideo.

**New records** (Figure 7): Argentina: Catamarca (Cuesta del Totoral); Chaco (El Zapallar); Córdoba (Calamuchita, La Serranita); Corrientes (Santo Tomé); Mendoza (Nihuil); Misiones (Apostoles, Iguazú, Pindapoy, San Ingacio, San Javier, Santa María); Salta (Cabra Corral, Corralito, El Alisal, José de Metan). Brazil: Parana (Guaraniacú) [MLPA, Barriga-Tuñón, MACN].

**Epicauta (Epicauta) nigerrima (Dugès, 1870)**

*Cantharisnigerrima* Dugès, 1870: 161.

*Epicautanigerrima*: Dugès 1889: 77; Pinto 1991: 225.

*Epicautacorvina*: Horn 1885: 107; Champion 1892: 418; Werner 1945: 446.

**Terra typica.**Pinto (1991) indicates that three males in the Salle Collection (BMNH) are from León.

**Distribution.** Mexico.

**Location.** Mexico: Aguascaliente, Durango, Guanajuato, Jalisco, Michoacán, Nuevo León, Querétaro, Sinaloa.

**Epicauta (Epicauta) nigritarsis (LeConte, 1853)**

*Lyttanigritarsis* LeConte, 1853: 340.

*Cantharisnigritarsis*: Gemminger and Harold 1870: 2152.

*Epicautanigritarsis*: Horn 1873: 100; 1885: 111; Denier 1935: 158; Werner 1945: 438; 1949: 105; Dillon 1952: 396; Selander 1959: 211; Werner et al. 1966: 37; Arnold 1976: 23; Pinto 1977: 137; 1982: 403; 1991: 282; Adams and Selander 1979: 234; Pinto and Mayor 1986: 602.

*Epicautahesitata* Dilon, 1952: 398.

Epicauta (Epicauta) nigritarisis Dillon, 1952: 398.

**Terra typica.***E.nigritarsis* Lectotype female (designated by Werner 1945) Mexican Boundary (Texas); MCZC. *E.hesitata* Holotype male, Belton, Texas; TAMU.

**Distribution.** Mexico, United States.

**Location.** Mexico: Coahulia, Nuevo León, Tamaulipas. United States: Arizona, New Mexico, Oklahoma, Texas.

**Epicauta (Epicauta) normalis Werner, 1944**

*Epicautanormalis* Werner, 1944: 65 (in part); 1949: 95 (in part); Vaurie 1950: 2 (in part); Dillon 1952: 392; Hatch 1965: 105; Werner et al. 1966: 38 (in part); Lavigne and Pfadt 1966: 7; Church 1967: 756; Kumar et al. 1967: 23; Rodgers and Lavigne 1972: 19; Pinto 1975: 430; 1980: 58; 1991: 277.

**Terra typica.** Holotype male, Bridgeport, California; MCZC.

**Distribution.** Canada, United States.

**Location.** Canada: Alberta, Saskatchewan. United States: Arizona, California, Colorado, Idaho, Kansas, Montana, Nevada, New Mwxico, North Dakota, South Dakota, Texas.

**Epicauta (Epicauta) obesa (Chevrolat, 1835)**

*Lyttaobesa* Chevrolat, 1835: 81.

*Cantharisobesa*: Dugès 1870: 128; Gemminger and Harold 1870: 2152.

*Lyttamus* Haag-Rutenberg, 1880: 55.

*Epicautaobesa*: Dugès 1889: 66; Champion 1892: 424; Denier 1935: 158; Blackwelder 1945: 483; Werner 1949: 100; Pinto 1982: 403; 1991: 196.

*Epicautaauricomans* Champion, 1982: 424; Vaurie 1950: 28; Pinto 1982: 403.

*Epicautaficta* Werner, 1949: 100; Arnold 1976: 21; Hoebeke 1978: 14.

*Epicautapseudosolani* Dillon, 1952: 395.

**Terra typica.***E.obesa* two syntypes (?) Toulepeck (=Toulepec), Mexico; UZMH.

**Distribution.** Canada, Mexico, United States.

**Location.** Canada: Ontario. Mexico: Distrito Federal, Hidalgo, Jalisco, Michoacán, Morelos, Nuevo León, Puebla, Querétaro, San Luis Potosí, Tamaulipas, Veracruz. United States: Arkansas, Connecticut, Florida, Georgia, Illinois, Iowa, Massachusetts, Michigan, New Hampshire, New Mexico, North Carolina, New York, Ohio, Oklahoma, Pennsylvania, South Carolina, Texas, Wisconsin.

**Epicauta (Epicauta) occidentalis Werner, 1944**

*Epicautalemniscata*: Horn 1873: 100; Ortenburger and Hatch 1926: 144; Ingram 1927: 1; Ingram and Douglas 1932: 71; Horsfall 1943: 32 (in part); Douglas 1935: 686; Werner 1945: 463; Dillon 1952: 389.

*Epicautavittata*: Bruner 1891: 15; Cockerell and Harris 1925: 31; Tafenelli and Bess 1968: 51.

*Epicautaoccidentalis* Werner, 1944: 69; 1945: 465; Dillon 1952: 399; Adams and Selander 1979: 246; Blodgett and Higgins 1988: 1456; Ray et al. 1989: 187; Pinto 1991: 348.

Epicauta (Epicauta) lemniscata: MackSwain 1956: 60.

**Terra typica.** Holotype male, Cambridge, Nebraska; MCZC.

**Distribution.** United States.

**Location.** United States: Alanbama, Arkansas, Colorado, Georgia, Illinois, Indiana, Kansas, Kentucky, Louisiana, Mississippi, Missouri, Nebraska, Oklahoma, South Dakota, Tennessee, Texas.

**Epicauta (Epicauta) ocellata (Dugès, 1870)**

*Cantharisocellata* Dugès, 1870: 160; Horn 1885: 107.

*Epicautaocellata*: Champion 1892: 414; Denier 1935: 158; Blackwelder 1945: 483; Selander 1959: 211; Pinto 1980: 70; 1991: 278.

**Terra typica.** Neotype male (designated by Pinto, 1980) Atlixco, Mexico; CASC.

**Distribution.** Mexico.

**Location.** Mexico: Coahuila, Durango, Morelos, Oaxaca, San Luis Potosí.

**Epicauta (Epicauta) ochropus (Haag-Rutenberg, 1880)**

*Lyttaochropus* Haag-Rutenberg, 1880: 28.

*Epicautaochropus*: Blackwelder 1945: 483.

**Terra typica.** Type (s?) Brazil, presumably in ZSBS.

**Distribution.** Brazil, contry labeled only.

**Epicauta (Epicauta) oregona Horn, 1875**

*Epicautaoregona* Horn, 1875: 153; Denier 1935: 158; Werner 1945: 461; Parker and Wakeland 1957: 26; 1975: 432; 1965: 105; Werner et al. 1966: 34; Church 1967: 755; Rees 1973: 199; Pinto 1975: 432, 435; 1980: 42; 1991: 286.

**Terra typica.** Lectotype male (designated by Werner 1945) Oregon; MCZC.

**Distribution.** Canada, Unted States.

**Location.** Canada: Alberta, British Columbia, Manitoba, Saskatchewan. Unted States: Arizona, Colorado, Idaho, Montana, Nebraska, Nevada, New Mexico, Dakota, Utah, Washington.

**Epicauta (Epicauta) pardalis LeConte, 1866**

*Epicautapardalis* LeConte, 1866: 157 (as race of *E.maculata*); Horn 1873: 96 (as species); Cahmpion 1892: 414; Denier 1935: 158; Blackwelder 1945: 484; Werner 1944: 65; 1945: 443; Vaurie 1950: 21; Dillon 1952: 392; Selander 1954: 87; Werner et al. 1966: 40; Pinto 1975: 43; 1980: 50; Ray et al. 1989: 187.

Epicauta (Epicauta) pardalis: MacSwain 1956: 58; Pinto 1991: 278.

**Terra typica.** Lectotype male (designated by Werner 1945) Valley of the Gila; MCZC.

**Distribution.** Mexico, United States.

**Location.** Mexico: Aguascalientes, Chihuahua, Coahuila, Durango, Sonora. United States: Arizona, New Mexico, Texas.

**Epicauta (Epicauta) parvula (Haldeman, 1853)**

*Meloeparvus* Haldeman, 1852: 377; LeConte 1853: 329.

*Meloeparvulus* Haldeman, 1853: 404 (n. repl. name for *M.parvus* Haldeman, nec Solier, 1851).

*Namaspisparvulus*: Van Dyke 1928: 411.

*Epicautaparva*: Werner 1945: 449; Kumar et al. 1976: 23; Lavigne 1976: 754; Agafitei and Selander 1980: 13.

*Epicautaparvula*: Pinto 1991: 212.

**Terra typica.** Type (s) Santa Fe (Pinto 1991) inthicates that the type (s) species is unknown.

**Distribution.** United States.

**Location.** United States: Colorado, Kansas, Nebraska, New Mexico.

**Epicauta (Epicauta) pedalis LeConte, 1866**

*Epicautapedalis* LeConte, 1866: 157; Horn 1873: 99; 1885: 111; 1894: 356; Denier 1935: 158; Werner 1945: 440; 1949: 93–95; Pinto 1991: 255.

*Cantahrispedalis*: Gemminger and Harold 1870: 2152.

**Terra typica.** Lectotype male (designated by Werner 1945; erroneously as female) Cape San Lucas, Baja Calisfornia Sur, Mexico.

**Distribution.** Mexico.

**Location.** Mexico: Baja California Sur.

**Epicauta (Epicauta) pensylvanica (Degeer, 1775)**

*Cantharispensylvanica* Degeer, 1775: 15.

*Meloenigra* Woodhouse, 1800: 213.

*Lyttapensylvanica*: LeConte 1853: 339; 1854: 447.

*Lyttamorio* LeConte, 1854: 447.

*Cantharispennsylvanica*: Gemminger and Harold 1870: 2152.

*Epicautapensylvanica*: Riley 1877: 561; Champion 1892: 418; Wickham 1896: 34; Ulke 1902: 54; Chittenden 1903: 25; 1911: 92; Blatchely 1910: 1363; Gibson 1912: 83; Sherman 1913: 246; Rau and Rau 1916: 260; Milliken 1921: 6; Mutchler and Weiss 1924: 10, 18; Cocerell and Harris 1925: 31; Gowdey 1926: 13; Carruth 1931: 52; Staig 1940: 135; Horsfall 1914: 114; 1943: 13; Denier 1935: 158; Blackwelder 1945: 484; Vaurie 1950: 25; Dillon 1952: 388; Selander 1954: 24; 1981: 757; 1982: 156, 427; 1984: 2; Selander and Bouseman 1960: 202; Parker and Wakeland 1957: 26; Gupta 1965: 454; Werner et al. 1966: 33; Church 1967: 754; Mathwig 1968: 544; Selander and Weddle 1969: 36; Rees 1973: 200; Arnold 1976: 20; Kumar et al. 1976: 23; Lavigne 1976: 754; Adams and Selander 1979: 160, 172; Werner et al. 1980: 1405; McLain 1982: 396; Staines 1983: 47; Capinera et al. 1985: 1054; Goldburg 1987: 247; Lago and Mann 1987: 5; Blodgett and Higgins 1988: 1456; Ray et al. 1989: 187.

*Epicautapensylvanica*: Horn 1873: 102; Werner 1945: 447.

*Lyttaatrata*: Claypole 1881: 31.

*Epicautapotosina* Dugès, 1889: 89.

Epicauta (Epicauta) pensylvanica: MacSwain 1956: 47; Pinto 1991: 201.

**Terra typica.***E.pensylvanica*, *E.nigra* types unknown. *E.morio* Lectotype female (designated by Werner 1945) Texas; MCZC. *E.potosina* Holotype (?) Huasteca de San Luis Potosí, Mexico; UNAM.

**Distribution.** Canada, Mexico, United States.

**Location.** Canada: Alberta, Manitoba, New Brunswick, Ontario, Quebec, Sakatchewan. Mexico: Chihuahua, Coahuila, Durango, Nuevo León. United States: Alabama, Arizona, Arkansas, Colorado, Connecticut, Delaware, District of Columbia, Florida, Georgia, Idaho, Illinois, Indiana, Iowa, Kansas, Kentucky, Louisiana, Maine, Maryland, Massachusetts, Michigan, Minnesota, Mississippi, Missouri, Montana, Nebraska, New Hampshire, New Jersey, New Mexico, New York, North Carolina, North Dakota, Ohio, Oklahoma, Pennsylvania, Rhode Island, South Carolina, South Dakota, Tennessee, Texas, Utah, Vermont, Virginia, West Virginia, Wisconsin, Wyoming.

**Epicauta (Epicauta) peruensis Kaszab, 196**0

*Epicautaperuensis* Kaszab, 1960: 407; Pinto and Bologna 2016: 205.

**Terra typica.** Holotype male, Tarma, Paru; SUM.

**Distribution.** Peru.

**Location.** Peru: Mountain near Tarma 3300 m; Junín.

**Epicauta (Epicauta) philaemata (Klug, 1825)**

*Lyttaphilaemata* Klug, 1825: 434.

*Cantharisphilaemata*: Gemminger and Harold 1870: 2152.

*Epicautaphilaemata*: Borchmann 1917: 69, Denier 1935b: 158, Blackwelder 1945: 484, Adams and Selander 1979: 259.

**Terra typica.** Syntype (s?) Brazil; ZMHU location unknown.

**Distribution.** Argentina, Brasil, Venezuela.

**Location.** Argentina: Misiones, Tucumán. Brazil: Río de Janeiro, São Paulo, Santa Catarina. Venezuela: country labeled only.

**New records** (Figure 7): Argentina: Misiones (Aristóbulo del Valle, Arroyo Yacutinga, Dos de Mayo) [MACN].

**Epicauta (Epicauta) phoenix Werner, 1944**

*Epicautaphoenix* Werner, 1944: 66; 1945: 443; Werner et al. 1966: 39; Pinto 1980: 55; 1991: 279.

**Terra typica.** Holotype male, Phoenix, Arizona; MCZC.

**Distribution.** United States.

**Location.** United States: country labeled only.

**Epicauta (Epicauta) pilme Molina, 1758**

*Epicautapilme* Molina, 1758.

*Epicautalangei* Borchmann, 1930: 95; Denier 1935b: 156; Blackwelder 1945: 483; Di Iorio 2004: 168; Campos-Soldini et al. 2009: 4 **New Synonymy**.

*Epicautanigripes* Borchmann, 1930: 91; Denier 1935b: 158; Blackwelder 1945: 483; Di Iorio 2004: 170 **New Synonymy**.

**Diagnosis.** The species *E.langei* and *E.nigripes* are phentically very similar to *Epicautapilma* by the combination of the following characters: body length 7–14mm. Tegument black. Legs with femora orange with apex black, tibiae and tarsi black. Body vestiture black, setae short, erect and minimal (6–9 setae along one mm long line) allowing to see tegument. Head subrectangular. Antenna subcylindrical, tapering to apex; ratios length (L_Ant_) vs. width (W_Ant_) of antennomere female (L_Ant_/W_Ant_): 1.5 (I), 1.3 (II), 1.6 (III), 1.2 (IV), 1 (V-VII), 2.5 (VIII-X) 3.5 (XI); male: 1.6 (I), 0.8 (II), 2 (III), 1.3 (IV-X), 2.6 (XI). Pronotum subcampaniform. For this reason, we consider these species as a single specific entity whose valid name is *E.pilma* by priority.

**Terra typica.***E.nigripes*, Syntypes, Catamarca, Valle de Santa María; MCNBR. *E.langei*, Type (s?) Catamarca, La Rioja; MCNBR.

**Distribution.** Argentina and Chile.

**Location.** Argentina: Buenos Aires, Catamarca, Chubut, Mendoza, Neuquén, Río Negro, Salta. Chile: Antofagasta, Arica, Atacama, Aysén, Bío Bío, Cautín, Coaquimbo, Chiloe, Lonquihue, Maule, O’Higgins, Parincota, Tarapacá, Valdivia, Valparaiso, Villa Rica.

**New records** (Figure 7): Argentina: Buenos Aires (Sierra de la Ventana); Chubut (El Ojo del Epuyen); Mendoza (Godoy Cruz, Las Heras); Neuquén (Huiliche, Lago Lacar, Parque Nacional Lanin, Perdriel, Pino Hachado, Pucará, Rinconada, San Martín de los Andes); Río Negro (Bariloche, El Bolsón, Chimpay) Salta (Cachipampa, Cuesta del Obispo, Isonza). Chile: Antofagasta; Arica; Atacama; Aysén; Bío Bío; Cautín (Temuco); Coaquimbo; Chiloe; Lonquihue; Maule; O’Higgins, Parincota; Santiago de Chile; Tarapacá; Valdivia; Valparaiso; Villa Rica (Lago Caburgua) [MLPA, IADIZA, Barriga-Tuñón, FIMLA, MACN].

**Epicauta (Epicauta) pluvialis Borchmann, 1930**

*Epicautapluvialis* Borchmann, 1830: 95; Denier 1935b 158; Bosq 1943: 11; Blackwelder 1945: 484; Viana and Williner 1974: 11; Martínez 1992: 7; Di Iorio 2004: 171; Campos-Soldini and Roig-Juñent 2015: 33.

**Terra typica.** Type (s?) Buenos Aires and Mendoza; MCNBR.

**Distribution.** Argentina.

**Location.** Argentina: Buenos Aires, Catamarca, Córdoba, Chubut, Entre Ríos, La Pampa, La Rioja, Mendoza, Salta, San Juan, San Luís, Río Negro.

**New records** (Figure 7): Argentina: Buenos Aires (Miramar); Catamarca (Hualfín, Punta Balasto, Santa María); Chubut (Península de Valdés); Córdoba (Ascochinga, Los Cocos); Entre Ríos (Victoria); Mendoza (Barrancas, El Azufre, Challao, La Cuenca, La Agüadita, Las Heras, Lujan del Cuyo, Malargüe, Potrerillo, San Carlos Estrada, San Rafael, Villavisencio); Neuquén (Las Lajas); Río Negro (Choele Choel, Río Colorado); San Luis (Coralina) [IADIZA, Barriga-Tuñón, FIMLA, MACN].

**Epicauta (Epicauta) proscripta Pinto, 1980**

*Epicautaproscripta* Pinto, 1980: 71; 1991: 279.

**Terra typica.** Holotype male, Jackson, Mississippi; USNM.

**Distribution.** United States.

**Location.** United States: Kansas, Mississippi, Tennessee.

**Epicauta (Epicauta) pruinosa LeConte, 1866**

*Epicautapruinosa* LeConte, 1866: 158; Horn 1873: 98; Maybell 1934: 327; Denier 1940: 422; Werner 1945: 471; Hatch 1965: 106; Werner et al. 1966: 44; Pinto 1991: 317.

*Lyttaimmertia* Walker, 1866: 330.

*Cantharispruinosa*: Gemminger and Harold 1870: 2153.

*Epicautapiceiventris* Maydell, 1934: 327; Werner 1945: 472; Hatch 1965: 106.

*Epicautaelongatocalcarata* Maydell, 1934: 328.

*Epicautalupini* Selander, 1952: 131.

*Epicautawerneri* Hatch, 1965: 105.

**Terra typica.***E.pruinosa*, Lectotype female (designated by Werner 1945) Colorado Territory; MCZC. *E.immerita*, Holotype female, British Columbia; BMNH. *E.piceiventris*, Holotype male, Utah; MCZC. *E.elongatocalcarata*, Holotype female, Atlanta, Idaho; MCZC. *E.lupini*, Holotype male, Tygh Valley, Oregon; USNM. *E.werneri*, Holotype female

**Distribution.** Canada, United States.

**Location.** Canada: Alberta, British Columbia, Manitoba. United States: Arizona, California, Colorado, Idaho, Montana, Nebraska, New Mexico, North Dakota, Oregon, South Dakota, Utah, Washington, Wyoming.

**Epicauta (Epicauta) pullata (Berg, 1889)**

*Lytta*pullata Berg, 1889: 121; Bruch 1914: 405; Borchmann 1917: 97.

*Epicautapullata*: Denier 1935b: 158; Blackwelder 1945: 484; Campos-Soldini et al. 2009: 5.

**Terra typica.** Syntypes (sex?) Chacabuco, Mendoza.

**Distribution.** Argentina.

**Location.** Argentina: Buenos Aires, Mendoza.

**New records** (Figure 7): Argentina: Buenos Aires: Chacabuco; Mendoza (Province labeled omly) [MLPA].

**Epicauta (Epicauta) puncticollis Mannerheim, 1843**

*Epicautapuncticollis* Mannerheim, 1843: 288; LeConte 1851: 162; Horn 1873: 97; 1885: 111; Gibson 1912: 87; Wickham 1914: 492; Maydell 1934: 327; Denier 1935: 158; Moore 1937: 42; Werner 1945: 474; Parker and Wakeland 1957: 27; Middlekauff 1958: 9; Gupta 1965: 453; Hatch 1965: 106; Church 1967: 757; Pinto 1973: 968; 1975: 451; Rees 1973: 202; Cohen and Pinto 1977: 741; Ballmer 1980: 78; Pinto and Mayor 1986: 602; Pinto 1991: 333.

*Cantharispuncticollis*: Gemminger and Harold 1870: 2153.

*Epicautaoblita*: LeConte 1853: 339; Horn 1873: 97; 1885: 111; Fall 1901: 184; Maydell 1934: 327; Moore 1937: 41; Rees 1973: 198; Pinto1977: 141; Werner 1945: 475.

*Lyttaoblita*: LeConte 1853: 339.

*Lyttapuncticollis*: LeConte 1853: 339.

*Epicautabarberi*: Werner 1944: 69; 1945: 475.

Epicauta (Epicauta) oblita: MacSwain 1956: 64.

Epicauta (Epicauta) puncticollis: MacSwain 1956: 62; Pinto 1974: 5.

**Terra typica.***E.puncticollis*, Holotype male, “Calif. Bor.”; UZMH; *E.oblita* Lectotype female (designated by Werner 1945) San Francisco, California; MCZC. *E.barberi*, Holotype male, La Panza, California; USNM.

**Distribution.** México, United States.

**Location.** Mexico: El Sauzi, Ensenada, San Antonio del Mar, Santo Tomás. United States: British Columbia, California, Montana, Nevada, San Francisco, Utah, Wayoming.

**Epicauta (Epicuta) punctipennis Werner, 1944**

*Epicautapunctipennis* Werner, 1944: 68; Dillon 1952: 391; Pinto 1975: 437; 1991: 119.

*Epicautapallidilabra* Dillon, 1952: 390.

**Terra typica.** Holotype male, Victoria, Texas; USNM.

**Distribution.** Canada, United States.

**Location.** Canada: British Columbia east to Manitoba. United States: Missouri, Nevada, New Mexico, South Dakota, Washington.

**Epicauta (Epicauta) purpureiceps (Berg, 1889)**

*Lyttapurpureiceps* Berg, 1889: 123; Bruch 1914: 405; Borchmann 1917: 97.

*Cantharispurpureiceps*: Chmapion 1899: 186.

*Epicautapurpureiceps*: Borchmann 1917: 97.

Epicautakraussivar.purpureiceps: Denier 1935: 156; 1940: 156.

**Terra typica.** Holotype female (?) Cordoba, Argentina; MLPA.

**Distribution.** Argentina, Brazil.

**New records** (Figure 7): Argentina: Córdoba, Santa Fe (provinces labeled only) [MLPA].

**Epicauta (Epicauta) rehni Maydell, 1934**

*Epicautarehni* Maydell, 1934: 329; Denier 1940: 422; Werner 1945: 485; 1949: 107; 1955: 5; Werner et al. 1966: 41; Pinto 1972: 254; 1991: 153.

**Terra typica.** Holotype female, Schaeffer Canyon, Bobaquivari Mts., Arizona; ANSP.

**Distribution.** United States.

**Location.** United States: Arizona, New Mexico.

**Epicauta (Epicauta) rileyi Horn, 1874**

*Epicautarileyi* Horn, 1874: 43; 1891: 43; Van Dyke 1929: 129; Maydell 1934: 329, 330; Denier 1935: 159; Pinto 1991: 154.

**Terra typica.** Lectotype female: (designated by Werner 1945) Arizona; MCZC.

**Distribution.** Mexico, United States.

**Location.** Mexico: Sonora. United States: Arizona.

**Epicauta (Epicauta) riojana (Fairmaire, 1892)**

Cantharisgriseonigravar.riojana Fairmaire, 1892: 252.

Lyttagriseonigravar.riojana: Bruch 1914: 405.

Epicautagriseonigravar.riojana: Borchmann 1917: 76; Denier 1935b: 156; Blackwelder 1945: 483.

*Epicautariojana*: Campos-Soldini 2011: 582.

**Terra typica.** Unknown.

**Distribution.** Argentina.

**Location.** Argentina: Córdoba; Entre Ríos, La Rioja, Salta, Tucumán.

**New records** (Figure 8): Argentina: Córdoba (Mansilla); Entre Ríos (Villa Elisa); Salta (Santa Salta Forestal); Tucumán (Gobernador Garmendia) [IADIZA, Barriga-Tuñón].

**Epicauta (Epicauta) rubella Denier, 1940**

*Epicautarubella* Denier, 1940: 182; Blackwelder 1945; Campos-Soldini et al. 2009: 6; Campos-Soldini and Roig-Juñent 2015: 35.

**Terra typica.** Holotype male, Paraguay: Puerto Max; MLPA. Allotype female, Salta: Esteco (MLPA).

**Distribution.** Argentina, Paraguay.

**Location.** Argentina: Salta: Departamento Salta Forestal. Paraguay: Puerto Max.

**Epicauta (Epicauta) rufipennis** (Chevrolat, 1834)

*Canthrisrufipennis* Chevrolet, 1834: 80; Dugès 1870: 127.

*Cantharisocreaceipennis* Dugès, 1870: 164.

*Cantharisrufipennis*: Gemminger and Harold 1870: 2153.

*Cantharisochreipennis* Dugès, 1877: 57.

*Epicautarufipennis*: Dugès 1889: 60; Champion 1892: 408; Denier 1935; 159; Blackwelder 1945: 484; Pinto 1991: 256.

**Terra typica.***E.rufipennis* Holotype (sex?) Mexico; probably in UZMH.

**Distribution.** Guatemala, Mexico.

**Location.** Guatemala: Guatemala, Purulha, Baja Vera Paz. Mexico: Chihuahua, Distrito Federal, Guanajuato, Guerrero, Hidalgo, Jalisco, Mexico, Michoacán, Morelos, Nayarit, Oaxaca, Puebla, Sinaloa, Veracruz.

**Epicauta (Epicauta) rutilifrons Borchmann, 1930**

*Epicautarutilifrons* Borchmann, 1930: 91; Blackwelder 1945: 484; Martínez 1992: 8; Campos-Soldini et al. 2009: 6; Campos-Soldini and Roig-Juñent 2015: 40.

**Terra typica.** Syntypes, Ledesma, Jujuy and Senillosa; MACN.

**Distribution.** Argentina, Brazil.

**Location.** Argentina: Jujuy, Salta. Bolivia. Brazil: Matto Grosso.

**New records**: Bolivia (country labeled only) (FIMLA, MACN).

**Epicauta (Epicauta) sanguinicollis (LeConte, 1853)**

*Lyttasanguinicollis* LeConte, 1853: 344.

*Cantharissanguinicollis*: Gemminger and Harold 1870: 2153.

*Epicautasanguinicollis*: Horn 1873: 103; 1874: 37; Denier 1935: 159; Werner 1945: 473; Selander 1984: 3; Pinto 1991: 334.

**Terra typica.** Neotype female (designated by Pinto 1991) Florida; MCZC.

**Distribution.** United States.

**Location.** United States: Florida, Georgia, South Carolina.

**Epicauta (Epicauta) sanguinithorax (Haag-Rutenberg, 1880)**

*Lyttasanguinithorax* Haag-Rutenberg, 1880: 34.

*Epicautasanguinithorax*: Borchmann 1917: 82.

**Terra typica.** Holotype male, Peru; ZSBS.

**Distribution.** Argentina, Peru.

**Location.** Argentina: La Rioja. Peru.

**New records** (Figure 8): Argentina: La Rioja (Guayapa) (FIMLA).

**Epicauta (Epicauta) saopaoloana Kaszab, 1960**

*Epicautasaopaoloana* Kaszab, 1960: 285.

**Terra typica.** Allotype and Paratype female, São Paulo, Brazil; Sammlung des Museums G. Frey. Holotype male, Quimadas, Brazil; SUNM.

**Distribution.** Brazil.

**Location.** Brazil: São Paulo.

**Epicauta (Epicauta) semivittata (Fairmaire, 1875)**

*Cantharissemivittata* Fairmaire, 1875: 200.

*Cantharishemigramma* Mäklin, 1875: 632.

*Cantharisvirgata* Burmeister, 1881: 25.

*Epicautasemivittata*: Bruch 1914: 404; Borchmann 1917: 82; Denier 1935b: 159; Bosq 1934: 327; 1943: 12; Blackwelder 1945: 484; Viana and Williner 1974: 16; Di Iorio 2004: 171; Campos-Soldini and Roig-Juñent 2015: 40.

*Pyrotavirgata*: Borchmann 1917: 69.

**Terra typica.** Unknown.

**Distribution.** Argentina, Chile, Uruguay.

**Location.** Argentina: Buenos Aires, Catamarca, Córdoba, Corrientes, Jujuy, La Pampa, Mendoza, Santa Fé, San Luis, Tucumán. Uruguay: Carrasco.

**New records** (Figure 8): Argentina: Buenos Aires (province labeled only); Córdoba (Calamuchita, El Sauce) [Barriga-Tuñón, MACN].

**Epicauta (Epicauta) senilis Werner, 1949**

*Epicautasenilis* Werner, 1949: 102; Werner et al. 1966: 44; Pinto 1991: 155.

*Epicautacandidata*: Dillon 1952: 395.

**Terra typica.** Holotype male, Luna Co., New Mexico Pinto (1991) indicates that the type is unknown.

**Distribution.** Mexico, United States.

**Location.** Mexico: Chihuahua, Durango. United States: Arizona, New Mexico, Texas.

**Epicauta (Epicauta) sericans LeConte, 1866**

*Lyttasartorii* Haag-Rutenberg, 1880: 56.

*Epicautasericans* LeConte, 1866: 158; Horn 1873: 98; 1891: 43; Blair 1921: 281; Milliken 1921: 6; Carruth 1931: 52; Denier 1935: 159; Werner 1945: 469; Vaurie 1950: 29; Dillon 1952: 385; Parker and Wakeland 1957: 26; Burke 1963: 53; Werner et al. 1966: 44; Church 1967: 756; Goeden 1971: 42; Rees 1973: 205; Arnold 1976: 26; Selander 1952: 131; 1982: 427; Capinera et al. 1985: 1054.

*Cantharissericans*: Gemminger and Harold 1870: 2153.

Epicauta (Epicauta) callosa: MacSwain 1956: 56.

**Terra typica.***E.sericans*, Lectotype female (designated by Werner 1945) Kansas; MCZC. *E.sartorii*, types from “Mexico, Mirador”, location of syntypes is unknown.

**Distribution.** Canada, Mexico, United States.

**Location.** Canada: Alberta, Saskatchewan. Mexico: Chihuahua, Coahuila, Durango, Hidalgo, Jalisco, Mexico, Nuevo León, Puebla, Querétero, San Luis Potosí, Tamaulipas, Veracruz. United States: Alabama, Arkansas, Colorado, Georgia, Indiana, Iowa, Kansas, Kentucky, Louisiana, Minnesota, Missouri, Montana, Nebraska, New Mexico, North Dakota, Oklahoma, South Dakota, Tennessee, Wyoming.

**Epicauta (Epicauta) singularis Champion, 1892**

*Epicautasingularis* Champion, 1892: 427; Denier 1935: 159; Blackwelder 1945: 484; Vauri 1950: 30; Werner 1955: 5; Pinto 1982: 407; 1991: 157.

**Terra typica.** Lectotype male (designated by Pinto 1982) Monterrey, Nuevo León; BMNH.

**Distribution.** Mexico, United States.

**Location.** Mexico: Nuevo León, Texas.

**Epicauta (Epicauta) solaniperda Denier, 1940**

*Epicautasolaniperda* Denier, 1940: 422.

**Terra typica.** Holotype male and Allotype female, Huancayo, Peru; MLPA type locality unknown.

**Distribution.** Peru.

**Epicauta (Epicauta) straba Horn, 1891**

*Epicautastraba* Horn, 1891: 42; Fall 1901: 184; Van Dayke 1929: 129; Denier 1935: 159; Moore 1937: 42; Werner 1945: 483; 1955: 5; Pinto 1972: 254; 1991: 158.

**Terra typica.***E.straba* Lectotype (designated by Werner 1945) San Bernardino California; MCZC. *E.foxi* Holotype female, Jacumba, San Diego Co., California.

**Distribution.** Mexico, United States.

**Location.** Mexico: Baja California Norte. United States: California.

**Epicauta (Epicauta) strigata (Gyllenhal, 1817)**

*Lyttastrigata* Gyllenhal, 1817: 18.

*Cantharisstrigata*: Fischer 1827: 19.

*Epicautastrigata*: Borchmann 1917: 83; Martínez 1955: 58.

**Terra typica.** Type (s?) from Brazil; ZMU, type loction unknown.

**Distribution.** Brazil.

**Epicauta (Epicauta) strigosa (Gyllenhal, 1817)**

*Lyttastrigosa* Gyllenhal, 1817: 18; LeConte 1853: 314.

*Cantharisstrigosa*: Fischer 1827: 19.

*Cantharisnigricornis* Melsheimer, 1846: 53.

*Cantharisstrigosa*: Gemminger and Harold 1870: 2154.

*Epicautastrigosa*: Horn 1873: 97; 1875: 153; Blatchely 1910: 1361; Ulke 1902: 54; Sherman 1913: 246; Mutchler and Weiss 1924: 9, 18; Denier 1935: 159; Werner 1945: 467; 1957: 97, 98; Dillon 1952: 384; Selander 1952: 131; 1984: 3; Staines 1983: 48; Pinto 1991: 341.

**Terra typica.***E.strigosa*, type (s?) presumably in ZUMU. *E.nigricornis* Lectotype (sex?) (designated by Werner 1945) Alabama; MCZC.

**Distribution.** United States.

**Location.** United States: Alabama, Connecticut, District of Columbia, Florida, Georgia, Louisiana, Massachusetts, Mississippi, New Jersey, New York, North Carolina, Rhode Island, South Carolina, Texas, Virginia.

**Epicauta (Epicauta) stuarti LeConte, 1868**

*Epicautastuarti* LeConte, 1868: 54; Horn 1873: 101; Maydell 1934: 333; Werner 1945: 462; 1955: 3,4; Selander 1954: 24; Werner et al. 1966: 42; Pinto 1991: 160.

**Terra typica.** Lectotype female (designated by Werner 1945) Ft. Union, New Mexico; MCZC.

**Distribution.** United States.

**Location.** United States: Arizona, Colorado, Kansas, Nebraska, New Mexico, Oklahoma, Texas, Wyoming.

**Epicauta (Epicauta) subatra Dugès, 1889**

*Epicautasubatra* Dgès, 1889: 72; Champion 1892: 421; Denier 1935: 159; Blackwelder 1945: 484; Pinto 1991: 205.

**Terra typica.** Holotype female, Mexico (Pinto 1991) indicates that the types is probably lost).

**Distribution.** Mexico.

**Location.** Mexico: Hidalgo, Nuevo León, Puebla.

**Epicauta (Epicauta) subvittata (Erichson, 1848)**

Lytta (Epicauta) subvittata Erichson, 1848: 566.

*Lyttasublineata*: Haag-Rutenberg 1880: 53.

*Epicautasubvittata*: Borchmann 1917: 84.

**Terra typica.** Type (s?) Guayana (=British Guaiana); ZMHU, type location unknown.

**Distribution.** Brazil, Guayana.

**Location.** Brazil: São Paulo.

**New records** (Figure 8): Brazil: São Paulo (state labeled ony) [MLPA].

**Epicauta (Epicauta) suturalis (Haag-Rutenberg, 1880)**

*Lyttaalbicincta* Haag-Rutenberg, 1880: 23. **New Synonymy**.

*Cantharissuturalis* Germar, 1821: 247; Gemminger and Harold 1870: 2154.

*Epicautasuturalis*: Bruch 1911: 405; Borchmann 1917: 84; Denier 1935: 159; Blackwelder 1945: 484.

*Epicautaalbicincta*: Borchmann 1917: 70; Denier 1935: 152; Blackwelder 1945: 482.

**Diagnosis.***Epicautasutrualis* is phenetically alike to *E.albicincta* by the combination of the following characters: body length large (> 17mm). Tegument black. Body vestiture cinereous, setae short, decumbent, dense (28–34 setae along one mm long line). Antenna flattened dorsoventraly in both sexes; ratios length (L_Ant_) vs. width (W_Ant_) of antennomere (L_Ant_/W_Ant_) in female: 3.75 (I), 2.33 (II), 6 (III), 4.6 (IV) 4 (V-X) 4.3 (XI); in male 3 (I), 2 (II), 5 (III), 3.3 (IV), 3 (V-VIII), 5 (IX-XI).

**Terra typica.** Type (s?) Merida, Venezuela; ZSBS.

**Distribution.** Argentina, Bolivia, Brazil, Paragauay, Venezuela, Uruguay.

**Location.** Argentina: Buenos Aires, Misiones, Jujuy, Salta, Tucumán. Chile: Valparaiso. Bolivia: Chiquitos; Cuatro Ojos. Brazil: Campo Bello; Goyaz (Río Verde, Yathay); Matto Grosso; Río de Janeiro; São Paulo. Paraguay: Asunción. Uruguay: Canelones (La Floresta). Uruguay: Cerro Largo.

**New records** (Figure 8): Argentina: Buenos Aires (Cerro Largo); Misiones (Alto Paraná, Puerto Victoria); Sante Fe (Casilda, Rosario); Tucumán. Chile: Valparaiso (province labeled only). Bolivia: Santa Cruz (Chiquitos, Cuatro Ojos). Brazil: Goyaz (Río Verde,); Mato Grosso (state labeled only); Río de Janeiro (Río de Janeiro). Paraguay: Asunción. Uruguay: Cerro Largo (Cuhilla de Melo) [MLPA, FMILA, MACN].

**Epicauta (Epicauta) talpa (Haag-Rutenberg, 1880)**

*Lyttatalpa* Haag-Rutenberg, 1880: 32.

*Cantharistalpa*: Berg 1881: 306.

*Epicautatalpa*: Beauregard 1890: 511; Bruch 1911: 404; Borchmann 1917: 85; Denier 1935: 160; Blackwelder 1945: 484; Martínez 1992: 8.

**Terra typica.** Type (s?) Cordoba; presumably in ZSBS.

**Distribution.** Argentina, Bolivia, Brazil, Paraguay, Uruguay.

**Location.** Argentina: Chaco, Córdoba, Corrientes, Misiones, Salta, Santiago del Estero. Bolivia: Andrés Ibáñez (Santa Cruz de la Sierra). Brazil: Mato Grosso (Rancho Grande, San Antonio das Barras, Utiariti); Paraná (Prudentrópolis). Paraguay: Concepción (Río aquidaban); San Pedro (Cororo). Uruguay: Tacuarembó (San Gregorio de Polanco).

**New records** (Figure 8): Argentina: Córdoba (Alta Gracia, Cruz del Eje, La Merced); Corrientes (Ituzaingó); Misiones (Dos de Mayo); Salta (Capiazutí, La Viña); Santiago del Estero (Río Salado). Bolivia: Andrés Ibáñez (Santa Cruz de la Sierra). Brazil: Mato Grosso (San Antonio das Barras, Utiariti); Parana (Prudentópolis). Paraguay: San Pedro (Cororo). Uruguay: Tacuarembó (San Gregorio de Polanco) [MLPA, MACN].

**Epicauta (Epicauta) tamara Adams & Selander, 1979**

*Epicautadugesi*: Werner, 1957: 107.

*Epicautatamara* Adams & Selander, 1979: 246; Pinto 1991: 349.

**Terra typica.** Holotype male, Culicán, Sinaloa, Mexico; AMNH.

**Distribution.** Mexico.

**Location.** Mexico: Sinaloa, Sonora.

**Epicauta (Epicauta) tarasca Pinto, 1991**

*Epicautatarasca* Pinto, 1991: 207.

**Terra typica.** Holotype male, 3 km E Quiroga, Michoacán, Mexico; CASC.

**Distribution.** Mexico.

**Location.** Mexico: Jalisco, Michoacán, Morelos, Oaxaca.

**Epicauta (Epicauta) temexa Adams & Selander, 1979**

*Epicautavittata*: Dugès 1889: 87.

*Epicautalemniscata*: Champion 1892: 415; Snow 1906: 149; Dillon 1952: 398 (in part).

*Epicautatemexa* Adams & Selander, 1979: 248; Ray et al. 1989: 187.

**Terra typica.** Holotype male, Pearsall, Frio Co., Texas; AMNH.

**Distribution.** Mexico, United States.

**Location.** Mexico: Coahuila, Nuevo León, San Luis Potosí, Tamaulipas, Veracruz. United States: Texas.

**Epicauta (Epicauta) tenebrosa Werner, 1949**

*Epicautapedalis*: Horn 1894: 356 (in part); Werner 1945: 440 (in part).

*Epicautatenebrosa* Werner 1949: 93; Werner et al. 1966: 38; Pinto 1991: 259.

**Terra typica.** Holotype male, Tucson, Arizona; MCZC.

**Distribution.** Mexico, United States.

**Location.** Mexico: Sonora. United States: Arizona.

**Epicauta (Epicauta) teresa Mathieu, 1983**

Epicauta (Epicauta) teresa Mathieu, 1983: 156.

*Epicautateresa*: Pinto 1984: 378; 1991: 268.

**Terra typica.** Holotype male, 10 km northesast of Cintalapa, Chiapas, Mexico; CASC.

**Distribution.** Mexico.

**Location.** Mexico: Chiapas, Oaxaca.

**Epicauta (Epicauta) tricostata (Werner, 1943)**

*Pleuropomphatricostata* Werner, 1943: 32; 1945: 426; Vaurie 1950: 39; Dillon 1952: 379; MacSwain 1956: 66; Werner et al. 1966: 54; Pinto 1973: 957; 1977: 136; Selander 1981: 780.

*Epicautatricostata*: Pinto 1984: 381; 1991: 231; Pinto and Mayor 1986: 602.

**Terra typica.** Holotype male, Presidio, Texas; USNM.

**Distribution.** Mexico, United States.

**Location.** Mexico: Coahuila, San Luis Potosí. United States: Arizona, New Mexico, Texas.

**Epicauta (Epicauta) tristis (Mäklin, 1875)**

*Cantharistristis* Mäklin, 1875: 630.

*Lyttalugubris* Haag-Rutenberg, 1880; Borchmann 1917: 95.

*Epicautatristis*: Beauregard 1890: 512; Denier 1935: 160; Blackwelder 1945: 484; Di Iorio 2004: 172.

*Epicautalugubris*: Denier 1935: 156; Blackwelder 1945: 483 (Nudum erat nomen)

*Epicautaluguberrima* Denier, 1935: 156 **New Synonymy.**

**Diagnosis.***Epicautatristis* and *E.lugubris* are alike regarding several features, such as: body length 10–12 mm. Tegument black; head with reddish patch on front. Superficial nonparallel sculpturing: head and pronotum punctuate. Body vestiture dorsally black, marginal and ventraly cinereous; seate dense (28–34 setae along one mm long line). Antenna similar in both sexes; ratios length (L_Ant_) vs. width (W_Ant_) antennomere (L_Ant_/W_Ant_): 2.3 (I), 1.6 (II), 3.5 (III-XI), in male 2.3 (I), 1.6 (II), 3.6 (III), 2.3 (IV-XI). For this reason, we consider these species (*E.tristis* and *E.lugubris*) as a single specific entity whose valid name is *E.tristis* by priority.

**Terra typica.***Lyttalugubris* Type (s?) Brazil; ZSBS.

**Distribution.** Argentina, Bolivia, Brazil.

**Location.** Argentina: Córdoba, Jujuy, Misiones, Mendoza, San Juan, San Luis, Salta. Bolivia: Santa Cruz de la Sierra. Brazil.

**New records** (Figure 9): Argentina: Salta (province labeled only) [FIMLA].

**Epicauta (Epicauta) unilineata Champion, 1892**

*Epicautaunilineata* Champion, 1892: 415; Denier 1935: 160; Blackwelder 1945: 484; Adams and Selander 1979: 250.

**Terra typica.** Lectotype male (designated by Admas and Selander 1979) San Jerónimo, Guatemala; location unknown.

**Distribution.** El Salvador, Guatemala, Mexico.

**Location.** El Salvador: La Unión, San Salvador, Santa Ana. Guatemala: Baja Verapaz, Guatemala. Mexico: Guerrero, Veracruz.

**Epicauta (Epicauta) ventralis Werner, 1945**

*Epicautaventralis* Werner, 1945: 444; Pinto 1975: 430; 1980: 48.

*Epicautanormalis*: Werner 1944: 65 (in part); 1945: 442 (in part); Hatch 1965: 105; Werner et al. 1966: 38 (in part).

**Terra typica.** Holotype male, Walsenburg, Colorado; FMNH.

**Distribution.** Canada, United States.

**Location.** Canada: Alberta, Saskatchewan. United States: Arizona, California, Colorado, Idaho, Kansas, Montana, Nebraska, Nevada, New Mexico, North Dakota, Oregon, South Dakota, Utah, Washington, Wyoming.

**Epicauta (Epicauta) vicina (Haag-Rutenberg, 1880)**

*Lyttavicina* Haag-Rutenberg, 1880: 27.

*Epicautavicina*: Borchmann 1917: 85; Denier 1935: 160; Blackwelder 1945: 484.

*Epicautawagneri* Pic, 1916: 10; Denier 1935: 160; Blackwelder 1945: 484 **New Synonymy**.

**Diagnosis.** The tegument color (pale brown with head and pronotum black); body vestiture dense (28–34 setae along one mm long line); and seate elongate, disheveled apparance and yellow of *E.vicina* are similar to *E.wagneri*. For this reason, we consider this species as a single specific entity whose valid name is *E.vicina* by priority.

**Terra typica.** Type (s?) Brazil; “Mus. Vind. and Haag-Rutenberg (in part); ZSBS.

**Distribution.** Argentina, Brazil, Paraguay.

**Location.** Argentina: Chaco; Corrientes; Entre Ríos; Misiones; Santiago del Estero. Brazil: Guaiaba. Paraguay: Villa Rica.

**New records** (Figure 9): Argentina: Entre Ríos (Concordia, Paraná); Corrientes (Chavarría); Misiones (Pindapoy); Santiago del Estero (Río Dulce). Paraguay: Guairá (Villa Rica) [MLPA].

**Epicauta (Epicauta) vidua (Klug, 1825)**

*Lyttavidua* Klug, 1825: 11.

*Causimaluctuosa* Dejean, 1837: 248.

*Causimalugubris* Dejean, 1837: 248.

*Cantharisvidua*: Burmeister 1881: 23; Berg 1881: 303; Gemminger and Harold 1870: 2155.

*Causimavidua*: Denier 1935: 161; Blackwelder 1945: 484; Di Iorio 2004: 165.

**Distribution.** Argentina, Bolivia, Brazil.

**Location.** Argentina: Córdoba, Corrientes, Misiones (General Belgrano, Puerto Aguirre, and Santa Ana). Bolivia. Brazil: Parana.

**New records** (Figure 9): Argentina: Córdoba (Salsacate); Misiones (Aristóbulo del Valle, Arroyo Cañapirú, Arroyo Uruguaí, Puerto Aguirre, Ruta Nac. 12, Santa Ana, Salto Encantado). Brazil: Parana (state labled only) [IADIZA, MLPA, FIMLA, MACN].

**Epicauta (Epicauta) vittata (Fabricius, 1775)**

Typical race

*Lyttavittata* Fabricius, 1775: 260.

*Meloevittatus*: Thunberg 1781–1791: 109.

*Meloechapmani*: Woodhouse 1800: 214.

*Cantharisvittata*: Gemminger and Harold 1870: 2155.

*Epicautavittata*: Horn 1873: 100; Denier 1935: 160; Staig 1940: 139; Werner 1945: 464; Admas and Selander 1979: 240; Pinto 1991: 352.

*Epicautalemniscata*: Putnam 1876: 173; Werner 1945: 463 (in part); Blodgett and Higgins 1988: 1456.

Epicauta (Epicauta) vittata: MacSwain 1956: 49.

Lemniscate race

*Lyttalemniscata* Fabricius, 1801: 79.

*Cantharislemnsicata*: Fischer 1827: 19.

*Epicautalemniscata*: Schwarz 1878: 464; Chitenden 1903: 115; Swingle and Mayer 1944: 141; Frost 1964: 140; Kirk 1969: 63 (in part).

*Epicautavittata*: Watson 1917: 64.

Epicauta (Epicauta) vittata: Kirk 1970: 61 (in part).

**Terra typica.***E.vittata*, Lectotype (sex?) (designated by Werner 1945) GUHC. *E.chapmani*, Types, Bucks Co., Pennsylvania (apparently destroyed). *E lemniscata*, Type male, “Carolina”; UZMH.

**Distribution.** Typical race: Canada, United States. Lemniscate race: United States.

**Location.** Typical race: Canada: Ontario, Quebec. United States: Alabama, Arkansas, Connecticut, Delaware, District of Columbia, Florida, Georgia, Illinois, Indiana, Iowa, Kansas, Kentucky, Louisiana, Maryland, Massachusetts, Michigan, Minnesota, Mississippi, Missouri, Nebraska, New Jersey, New York, North Carolina, Ohio, Oklahoma, Pennsylvania, Rhode Island, South Carolina, South Dakota, Tennessee, Virginia, West Virginia. Lemniscate race: United States: Florida, Georgia, South Carolina.

**Epicauta (Epicauta) vitticollis (Haag-Rutenberg, 1880)**

*Lyttavitticollis* Haag-Rutenberg, 1880: 52.

*Epicautacanoi* Dugès, 1889: 86.

*Epicautavitticollis*: Champion 1892: 414; Denier 1935: 160; Blackwelder 1945: 484; Adams and Selander 1979: 250; Pinto 1991: 353.

**Terra typica.***E.vitticollis*, Lectotype male, “Guatimal./Samml. Haag-Rutenberg/” (desgnated by Pinto 1991) ZSMC. *E.canoi*, Types, Veracruz, Mexico; apparently lost.

**Distribution.** Belize, Guatemala, Honduras, Mexico, Nicaragua.

**Location.** Belize: Corazal, Orange Walk, Toledo. Guatemala: Alta Verapaz, Petén. Honduras: Atlantida, Colón, Francisco Morazán, Yoro. Mexico: Chiapas, Oaxaca, Quintana Roo, Tabasco, Yucatán, Veracruz. Nicaragua: Río San Juan.

**Epicauta (Epicauta) weyrauchi Kaszab, 1960**

*Epicautaweyrauchi* Kaszab, 1960: 402. Pinto and Bologna 2016: 206.

**Terra typica.** Holotype and Paratype male, Andahuaylas, Peru. SMB.

**Distribution.** Peru.

**Location.** Peru: Apurimac.

**Epicauta (Epicauta) wheeleri Horn, 1873**

*Epicautawheeleri* Horn, 1873: 101, 107; Werner 1945: 486; 1949: 107; 1955: 5; Werner et al. 1966: 41; Pinto 1972: 256, 1973: 968; 1991: 161.

**Terra typica.** six syntypes, Arizona; MCZC.

**Distribution.** United States.

**Records.** United States: Arizona, California, Nevada, Utha.

**Epicauta (Epicauta) willei Denier, 1940**

*Epicautawillei* Denier, 1940: 422; Campos-Soldini et al. 2009: 6; Pinto and Bologna 2016: 206.

**Terra typica.** Holotype male, Allotype female, Paratypes male and female, Huancayo, Peru; MLPA.

**Distribution.** Peru.

**Location.** Peru: Cuzco: Sicuani, Huancayo and Junín.

**Epicauta (Epicauta) xanthomera (Fischer, 1827)**

*Cantharisxanthomeros* Fischer, 1827: 19; Geminnger and Harold 1870: 2155.

*Epicautaxanthomera*: Denier 1935: 169.

**Terra typica.** Unknown.

**Distribution.** Argentina, Brazil, Uruguay.

**Location.** Argentina: Córdoba, La Rioja. Brazil: Mina Gerais; Río de Janeiro. Uruguay: Cerro Largo.

**New records** (Figure 9): Argentina: Córdoba (Calamuchita, Unquillo); La Rioja (province labeled only). Brazil: Mina Gerais (Belo Horizonte); Río de Janeiro (state labeled only). Uruguay: Cerro Largo (Cañada de los Burros) [MLPA, MACN].

**Epicauta (Epicauta) xanthocephala (Klug, 1825)**

*Lyttaxanthocephala* Klug, 1825: 434.

*Cantharisxanthocephala*: Fischer 1827: 20.

*Epicautaxanthocephala*: Borchmann 1917; Adams and Selander 1979: 260.

**Terra typica.** Type (s?) Brazil; ZMHU, type locality unknown.

**Distribution.** Brazil.

**Location.** Brazil: Goiáz.

**New records** (Figure 9): Brazil: Goiáz (Jatahy, Río Verde) [MLPA].

**Epicauta (Epicauta) yungana Denier, 1935**

*Epicautayungana* Denier, 1935: 160; 1940: 422; Admas and Selander 1979: 260.

**Terra typica.** Holotype male, Coroico, 1300–1700 m, Nor Yungas, Bolivia; MLPA.

**Distribution.** Argentina, Bolivia, Brazil, Guayana.

**Location.** Argentina: Chaco; Córdoba; Misiones. Bogotá: Campobello. Bolivia: Coroico; La Paz; Puerto Aguirre. Brazil: Itatiaya. Guayana: Puerto Pariacabo.

**New records** (Figure 9): Argentina: Chaco (Resistencia); Misiones (Salto Iguazú). Bogotá: Campobello. Bolivia: Coroico, Puerto Aguirre. Brazil: Itaiaya. Guayana: Puerto Pariacabo [MLPA].

**Epicauta (Epicauta) zebra (Dohrn, 1876)**

*Canthariszebra* Dohrn, 1876: 411.

*Lyttaalbovittata* Haag-Rutenberg, 1880: 29.

*Cantharisalbovittata*: Burmeister 1881: 23; Berg 1881: 303.

*Epicautasomnolenta* Beauregard, 1890: 510; Bruch 1914: 404; Borchmann 1917: 83; Bosq 1934: 12, Viana and Williner 1974: 11.

*Epicautazebra*: Denier 1935b: 161; 1940: 422; Hayward 1942: 23; Blackwelder 1945: 484; Adams and Selander 1979: 162; Martínez 1992: 8; Di Iorio 2004: 172; Campos-Soldini 2011: 583.

**Terra typica.***Canthariszebra*, Type (s?) Córdoba, Argentina, present location unknown. *Lyttaalbovittata*, Syntypes Córdoba, Argentina; presumably in ZSBS.

**Distribution.** Argentina.

**Location.** Argentina: Catamarca, Córdoba, La Rioja, Mendoza, Misiones, Salta, San Juan, San Luis, Santiago del Estero, Tucumán.

**New records** (Figure 9): Argentina: Catamarca (Andalgalá); Córdoba (La Falda, La Paz, San Javier); La Rioja (Chilecito, Tinogasta); Mendoza (Desaguadero, Ñacuñan, Santa Rosa); Misiones (Santa María); San Luis (Balde, San Gerónimo) [IADIZA, Barriga-Tuñón; FIMLA, MACN]

**Epicauta (Epicauta) zischkai Martinez, 1955**

*Epicautazischakai* Martínez, 1955: 55; Kaszab 1960: 404; Di Iorio 2004: 172; Pinto and Bologna 2016: 206.

**Terra typica.** Holotype male, Allotype female, 15 Paratype male and nine Paratype female in FML. 15 Paratype male and three Paratype female in the Universidad de Vermont in Burlington, Vermont, USA. One Paratype (sex?) in Munich Muesum, Gemany.

**Distribution.** Bolivia, Peru.

**Location.** Bolivia Chapare, Cochabamba. Peru: Lago Titicaca, Puno.
